# The Role of the Anion in Imidazolium-Based Ionic Liquids for Fuel and Terpenes Processing

**DOI:** 10.3390/molecules28062456

**Published:** 2023-03-07

**Authors:** Aline Zambom, Sérgio M. Vilas-Boas, Liliana P. Silva, Mónia A. R. Martins, Olga Ferreira, Simão P. Pinho

**Affiliations:** 1 Centro de Investigação de Montanha (CIMO), Instituto Politécnico de Bragança, Campus de Santa Apolónia, 5300-253 Bragança, Portugal; 2 Laboratório para a Sustentabilidade e Tecnologia em Regiões de Montanha, Instituto Politécnico de Bragança, Campus de Santa Apolónia, 5300-253 Bragança, Portugal; 3 CICECO—Aveiro Institute of Materials, Complexo de Laboratórios Tecnológicos, University of Aveiro, Campus Universitário de Santiago, 3810-193 Aveiro, Portugal

**Keywords:** ionic liquids, terpenes, fuel processing, deterpenation, inverse gas chromatography

## Abstract

The potentialities of methylimidazolium-based ionic liquids (ILs) as solvents were evaluated for some relevant separation problems—terpene fractionation and fuel processing—studying selectivities, capacities, and solvent performance indices. The activity coefficients at infinite dilution of the solute (1) in the IL (3), $\gamma_{13}^{\infty }$, of 52 organic solutes were measured by inverse gas chromatography over a temperature range of 333.2–453.2 K. The selected ILs are 1-butyl-3-methylimidazolium hexafluorophosphate, [C_4_mim][PF_6_], and the equimolar mixture of [C_4_mim][PF_6_] and 1-butyl-3-methylimidazolium chloride, [C_4_mim]Cl. Generally, low polar solutes follow $\gamma_{1,\left[C_{4}\mathrm{mim}\right]\mathrm{Cl}}^{\infty }$ > $\gamma_{1,\left[C_{4}\mathrm{mim}\right]\left[{\mathrm{PF}}_{6}\right]+\left[C_{4}\mathrm{mim}\right]\mathrm{Cl}}^{\infty }$ > $\gamma_{1,\left[C_{4}\mathrm{mim}\right]\left[{\mathrm{PF}}_{6}\right]}^{\infty }$ while the opposite behavior is observed for alcohols and water. For citrus essential oil deterpenation, the results suggest that cations with long alkyl chains, such as ${\left[C_{12}\mathrm{mim}\right]}^{+}$, promote capacity, while selectivity depends on the solute polarity. Promising results were obtained for the separation of several model mixtures relevant to fuel industries using the equimolar mixture of [C_4_mim][PF_6_] and [C_4_mim]Cl. This work demonstrates the importance of tailoring the polarity of the solvents, suggesting the use of ILs with mixed anions as alternative solvents for the removal of aliphatic hydrocarbons and contaminants from fuels.

## 1. Introduction

Nowadays, many organic solvents commonly used in industrial separation processes are not compliant with the green chemistry principles [1] because of their substantial volatility, flammability, and toxicity [2]. For a solvent to be considered a ‘green solvent’, it should be stable (chemically and physically), easy to manage, recyclable, and have low volatility [3].

In the last decades, ionic liquids (ILs) have been proposed as green alternatives to traditional organic solvents due to their negligible volatilities associated with high thermal stabilities, which are properties that bring several advantages at an industrial scale. On a practical level, ILs are easier to store and generally safer due to their lower flammability [3,4]. 

Despite the many advantages of ILs over commonly used organic solvents, several studies have demonstrated their significant level of ecotoxicity [5]. Therefore, generalizations in terms of their green advantages should be avoided and each IL analyzed on a case-by-case basis [6]. Another disadvantage commonly referred to as a limitation in applying ILs to large-scale processes is their high cost of production; although, recent studies have introduced more cost-effective options [7,8]. ILs are salts, usually composed of organic cations and organic or inorganic anions, that can be tailored to have certain properties and are therefore known as designer solvents [9,10,11,12]. In general, the anionic structure has a greater influence on the ILs’ physical and chemical properties [10,13,14]. 

Due to their excellent solvation capabilities [11,15], ILs have been widely investigated as separation agents in several extraction processes such as fuel processing [11,12,16,17,18,19,20], separation of aromatic from aliphatic hydrocarbons [11,12,16,21,22,23,24], and terpenes fractionation [11,25]. Regarding fuel processing, one of the main constraints faced by industries is related to the removal of impurities present in fuel oils [18,26]. The research groups of Domańska [21,27], Mutelet [28,29], and Pinho [12,16] have been investigating ILs as alternatives to volatile organic compounds for the removal of aromatics from aliphatic hydrocarbons, as well as for the extraction of sulfur and nitrogen compounds from fuels. Vilas-Boas et al. [12], for example, evaluated the potential of [C_8_mim]Cl, [C_12_mim]Cl, and equimolar mixtures of [C_4_mim]Cl and [C_12_mim]Cl in the removal of aromatic hydrocarbons from aliphatics, the desulfurization and denitrification of fuels, and the separation of azeotropic mixtures containing alkanols, showing that cations with larger alkyl chains have more affinity with organic solutes. Likewise, eutectic mixtures have also been explored to separate common constituents of fuel mixtures [11,30,31], being promising options to be used as liquid media in different separation processes.

In the deterpenation context, the removal of terpenes hydrocarbons from essential oil (EO) rich in oxygenated terpenes (terpenoids) improves the quality and stability of the final product, which are important characteristics for the cosmetic, pharmaceutical, and food industries [32,33,34]. Martins et al. [25], and Vilas-Boas et al. [11] already studied imidazolium and phosphonium based ILs as entrainers for terpene fractionation, reporting that polar anions favor the separation of most mixtures containing terpenes.

This work is the continuation of our recent series [11,12,16,25,35] in which we aim to understand solute-IL interactions and to give tools for the appropriate choice of a solvent for a given separation problem. The activity coefficients at infinite dilution, $\gamma_{13}^{\infty }$, of 52 solutes (water, alkanes, cycloalkanes, ketones, ethers, cyclic ethers, aromatic hydrocarbons, esters, alcohols, terpenes, and terpenoids) in 1-butyl-3-methylimidazolium hexafluorophosphate, [C_4_mim][PF_6_], and in the equimolar mixture of [C_4_mim][PF_6_] and 1-butyl-3-methylimidazolium chloride, [C_4_mim]Cl, were measured by inverse gas chromatography technique over the temperature range of 333.2–453.2 K. Similar information was already reported in the literature [16,25] for [C_4_mim]Cl. From the $\gamma_{13}^{\infty }$ experimental values, some thermodynamic parameters such as gas-liquid partition coefficients, excess partial molar properties, selectivities, capacities, and solvent performance indices were calculated and discussed in order to evaluate the ILs’ performances in important separation processes. By selecting two ILs with a common cation, but different anions, the effect of changing the concentration of the anions was evaluated and the designer character of ILs explored.

## 2. Results

### 2.1. Activity Coefficients at Infinite Dilution

The activity coefficients at infinite dilution translate the solute (1)—solvent (3) affinities: $\gamma_{13}^{\infty }$ > 1, the solute–solute interactions are stronger than the solute–solvent ones; $\gamma_{13\;}^{\infty }$ = 1, similar solute–solute and solute–solvent interactions; $\gamma_{13}^{\infty }\;$< 1, the solute–solvent interactions are higher than the interaction between solute molecules. In this work, stationary phases of [C_4_mim][PF_6_], equimolar [C_4_mim][PF_6_]/[C_4_mim]Cl mixture, and [C_4_mim]Cl [16,25] were selected in order to explore the anion effect in the $\gamma_{13\;}^{\infty }$ and derived properties. The $\gamma_{13}^{\infty }$ of 52 solutes were measured by inverse-gas chromatography in the global temperature range of 333.2*–*453.2 K; those are listed in Table S3 and represented in Figure S1 of Section S3 of the Supplementary Materials. The lower temperature was chosen considering the pure IL’s melting point, and for each solute, at least three different temperatures were investigated.

To our best knowledge, experimental $\gamma_{13}^{\infty }$ data in the equimolar mixture are reported here for the first time, as well as the values of terpenes and terpenoids in [C_4_mim][PF_6_]. Common organic solutes were studied before in [C_4_mim][PF_6_] by several authors [28,36,37], and a comparison is available in Figure S2 for some solutes. Similar trends are found for most solutes, even though most literature data were measured in a lower temperature range. Overall, this comparison gives excellent indications about the consistency of our method and the data measured in this work.

**Figure 1:**
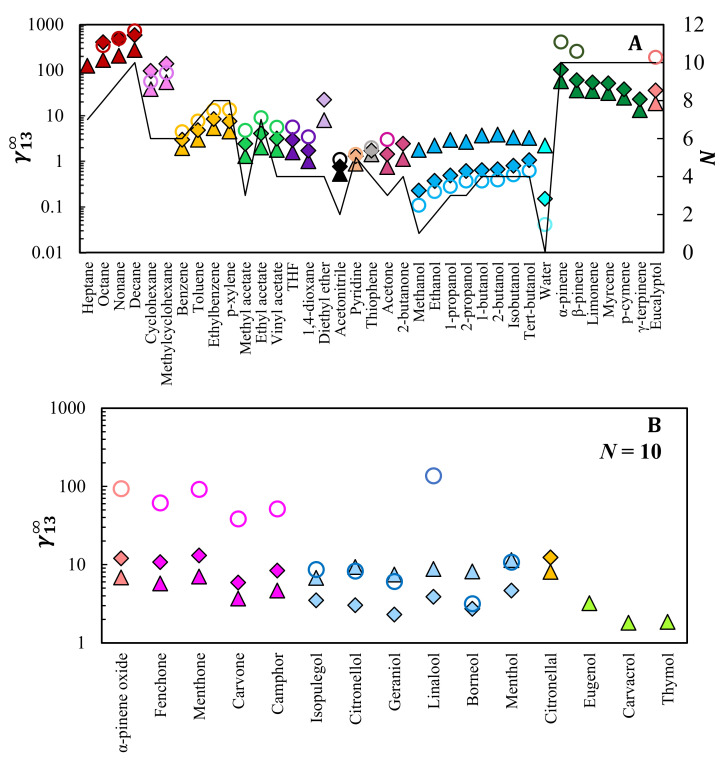
Activity coefficients at infinite dilution of several solutes in: (◆), [C_4_mim][PF_6_]/[C_4_mim]Cl equimolar mixture; (▲), [C_4_mim][PF_6_]; (○), [C_4_mim]Cl (from literature) [16,25]. Top panel, (**A**): traditional organic solutes, water, and some terpenes/terpenoids at 353.2 K. Bottom panel, (**B**): less volatile terpenoids at 413.2 K. Symbols with different colors represent different chemical families while the solid line indicates the number of carbons (*N*) present in each solute. On the bottom panel, all solutes have 10 carbon atoms.

Figure 1 shows the experimental $\gamma_{13}^{\infty }$, measured at 353.2 K and 413.2 K. The highest temperature was selected for the less-volatile terpenoids (bottom panel). Due to the long retention times of the phenolic terpenoids (eugenol, carvacrol, and thymol) and the TCD detector sensitivity limit, these solutes were not analyzed in the equimolar mixture. 

From a global analysis of Figure 1A, the $\gamma_{13}^{\infty }$ values for most solutes follow the solvent polarity, i.e., are higher in [C_4_mim]Cl (more polar) and in the equimolar mixture than in [C_4_mim][PF_6_] (less polar). Due to their strong polar character, alcohols and water are capable of hydrogen bonding, so their interactions are stronger with the more-polar chloride anion than with [PF_6_]^−^, which has a more shielded negative charge. This leads to an inverse trend, with $\gamma_{13}^{\infty }$ being lower than one (high solvent–solute affinity) for most alcohols and water in pure [C_4_mim]Cl or the equimolar mixture. By comparing the results of the equimolar mixture with those of pure [C_4_mim]Cl and [C_4_mim][PF_6_], it is possible to notice that, apart from alkanes, cycloalkanes, and diethyl ether, the equimolar mixture assumes intermedium values between the pure ILs. Yet, particularly for alcohols and water, the values for the equimolar mixture are closer to pure [C_4_mim]Cl than pure [C_4_mim][PF_6_], suggesting that the chloride anion has a greater influence on the $\gamma_{13}^{\infty }$. For alkanes: $\gamma_{13,\;\;equimolar\;mixture}^{\infty }\sim \gamma_{13,\;\;\left[C_{4}mim\right]Cl}^{\infty }>\gamma_{13,\;\;\left[C_{4}mim\right]\left[PF_{6}\right]}^{\infty },$ and for cycloalkanes: $\gamma_{13,\;\;equimolar\;mixture}^{\infty }>\gamma_{13,\;\;\left[C_{4}mim\right]Cl}^{\infty }>\gamma_{13,\;\;\left[C_{4}mim\right]\left[PF_{6}\right]}^{\infty }$. Due to their high volatility, however, this ordering presents a greater uncertainty. Comparing these results with the hydrocarbon terpenes, it seems that for the non-polar compounds the $\gamma_{13}^{\infty }$ values in the equimolar mixture are closer to pure [C_4_mim][PF_6_] than to [C_4_mim]Cl. Regarding the influence of the solute chain size, normally, for a given family, the $\gamma_{13}^{\infty }$ increase with the number of carbons in the chemical structure, reflecting the corresponding decrease in polarity. Activity coefficients at infinite dilution lower than unity were obtained for very polar solutes in polar ILs, such as alcohols, water, pyridine, and acetonitrile in [C_4_mim]Cl, indicating strong solute–solvent interactions. In particular, the $\gamma_{13}^{\infty }$ values for acetonitrile are lower than unity in all studied ILs, suggesting the high potential of these solvents to extract this probe from non-polar aliphatic hydrocarbons. Similarly, pyridine and thiophene also present low $\gamma_{13}^{\infty }$ values. As these compounds act as impurities in hydrocarbon mixtures, these results suggest high potential of the investigated ILs for the removal of nitrogen and sulfur-containing compounds from alkanes, as will be discussed later in this work. 

Regarding terpenes and terpenoids (Figure 1), all have a ten-carbon-atoms alkyl chain, so their apolar character results in $\gamma_{13}^{\infty }$ > 1. Nevertheless, some similar trends can be observed. The hydrocarbons α- and β-pinene show the weakest solute–IL affinity, namely in [C_4_mim]Cl which interacts better with polar solutes such as alcohol terpenoids. For the latter, the experimental results in [C_4_mim][PF_6_] are close to the literature data on [C_4_mim]Cl, apart from borneol and the outlier linalool. As mentioned by Martins et al. [25], linalool is the only tertiary alcohol among the studied terpenoids having a weaker H-bond acceptor character, resulting in higher $\gamma_{13}^{\infty }$, and consequently, lower solute–IL affinities. 

The temperature dependence of the ln($\gamma_{13}^{\infty }$.) as a function of 1⁄*T* is presented in Figure S1. A linear decrease*—*acetonitrile, pyridine, thiophene, acetone, 2-butanone, 1,4-dioxane, fenchone, eugenol, carvacrol and thymol*—*or increase*—*alkanes, cycloalkanes, diethyl ether, hydrocarbon terpenes, eucalyptol, and menthol—was observed for these systems in all the studied ILs. For other organic solutes, different trends were observed for the same solute in the different ILs, which is related to the corresponding affinities. Polar solutes as alcohols, water, and some alcohol terpenoids have a linear increase in polar solvents and a decrease in [C_4_mim][PF_6_]. Additionally, for ethyl acetate, THF, camphor, and menthone in [C_4_mim]Cl, the dependence with temperature is very small, meaning that the partial molar enthalpies are close to zero. This subject is investigated ahead.

### 2.2. Influence of the Anion Polarity

**Figure 2:**
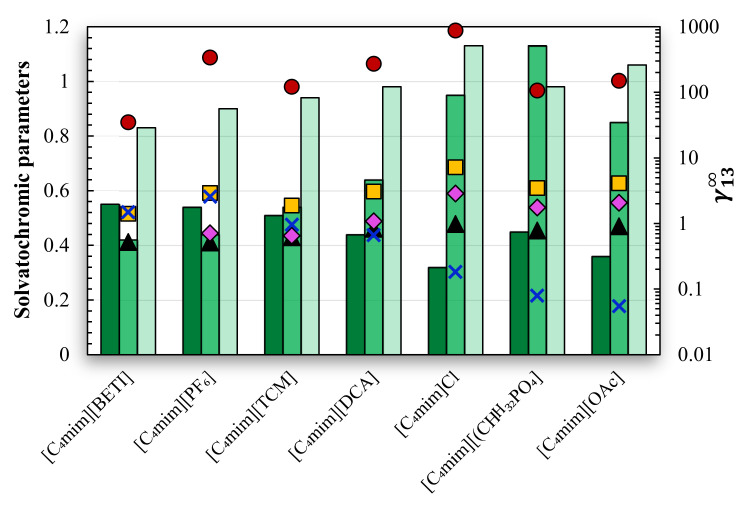
Activity coefficients at infinite dilution, $\gamma_{13}^{\infty }$, of: ●, decane; ■, toluene; ▲, acetonitrile; ◆, acetone; ✖, ethanol, at 333.2 K, in [C_4_mim][PF_6_] (this work), [C_4_mim][BETI] [41], [C_4_mim][TCM] [42], [C_4_mim][DCA] [17], [C_4_mim]Cl [16], [C_4_mim][(CH_3_)_2_PO_4_] [16], and [C_4_mim][OAc] [11]; and ILs’ solvatochromic parameters (bars): 🌢, α; 🌢, β; 🌢, π* [43,44].

To further evaluate the effect of the anion polarity in the solute–solvent interactions, the activity coefficients at infinite dilution of diverse solutes in an extended group of ILs, composed of different anions and a common cation (1-butyl-3-methylimidazolium), were compared (Figure 2). Additionally, the ILs’ solvatochromic parameters (Table S4), hydrogen bond acidity (α), hydrogen bond basicity (β), and dipolarity/polarizability (π*) were introduced as a measure of polarity [38,39,40].

To infer about the ILs’ hydrogen bond donor and acceptor abilities, acetone (hydrogen bond acceptor, HBA) and ethanol (hydrogen bond donor, HBD) probes were chosen. For the investigation of *σ*-electron and *π*-electron dispersion forces, decane and toluene, respectively, were selected. Finally, to explore the ability of the ILs to solvate dipole molecules, the ion + dipole interactions were studied using acetonitrile. 

As can be seen in Figure 2, in general, the $\gamma_{13}^{\infty }$ for the HBD solute (ethanol) decrease (higher solute–solvent affinity) with the increase of the IL polarity, in particular with the increase HBA ability of each anion measured by the Kamlet–Taft β parameter [44]. For acetone, the $\gamma_{13}^{\infty }$ decrease in the order Cl^−^ > [OAc]^−^ ≈ [(CH_3_)_2_PO_4_]^−^ > [DCA]^−^ > [PF_6_]^−^ ≈ [TCM]. Globally, it matches the trend where higher α corresponds to lower $\gamma_{13}^{\infty }$ values (higher the solute–IL affinity) [43,44,45,46]. Unlike the β parameter, α depends mainly on the cation’s nature [43,44,45,46], and that justifies the less-pronounced deviations between α and $\gamma_{13}^{\infty }$. when compared to β. Nonetheless, Figure 2 indicates that the anion also plays a significant role in the α values [44,45]. On the other hand, the anion polarity does not seem to influence the $\gamma_{13}^{\infty }$ of acetonitrile, which takes on similar values in all the studied ILs and is correlated to the π* values of the ILs. Concerning the *σ* and *π*-electron dispersion forces (decane and toluene), the highest and lowest $\gamma_{13}^{\infty }$ values are observed in [C_4_mim]Cl and [C_4_mim][BETI], respectively. The full trend is similar between both solutes and also to the trend observed for the π* values.

### 2.3. Gas–Liquid Partition Coefficients

The gas–liquid partition coefficients, $K_{L}$, of the solutes were calculated using Equation (S9) considering the solvent densities in Tables S5 and S6, and they are presented in Table S7 and Figure S3 of Section S3 of the SI. This thermodynamic parameter gives information about the distribution of a solute between the IL and the gas phase, providing insights into the suitability of a certain IL to act as a separation agent in a given industrial separation process [25].

As can be seen in Figure S3A, the number of carbons in the solute influenced $K_{L}$, namely for alkanes, cycloalkanes, aromatic hydrocarbons, acetates, ethers, ketones, and alcohols; where $K_{L}$ increases with the increase of the solute alkyl-chain. However, in the case of alcohols, the position of the hydroxyl group has great influence on the $K_{L}$. values. Primary alcohols such as 1-propanol and 1-butanol showed the highest values, followed by secondary alcohols (2-propanol, 2-butanol, and isobutanol). The tertiary alcohol studied (tert-butanol) showed the lowest $K_{L}$ values. Particularly for cyclic ethers, a higher number of oxygen atoms contributes to enhance the solute concentration in the liquid phase. For the nitrogen compounds (acetonitrile and pyridine), the values were similar in both ILs, being the highest in [C_4_mim][PF_6_] among all solutes. Overall, as the temperature increased, the $K_{L}$ decreased, as the solute concentration in the liquid phase is lower (Table S7). The $K_{L}$ values reported by Martins et al. [16] for alcohols are higher than those showed in [C_4_mim][PF_6_] due to the chloride anion capacity to form hydrogen bonds with these solutes. As expected, the opposite trend is observed for the nonpolar solutes. Considering the group of hydrocarbon terpenes, *p*-cymene had the highest $K_{L}$. value (314.04 in [C_4_mim][PF_6_]) and *α*-pinene had the lowest, closely followed by *β*-pinene. The slight difference can be explained by the change in the double-bond position in their structure, resulting in a stronger interaction between ILs and *β*-pinene. As expected, the less-volatile terpenes (Figure S3B) presented higher $K_{L}$ values. As in the $\gamma_{13}^{\infty }$ findings, the gas–liquid partition coefficients in [C_4_mim][PF_6_] reflect more favorable affinities with the more nonpolar solutes while [C_4_mim]Cl presents a higher solute–IL affinity with polar solutes. Concerning the $K_{L}$ values for the [C_4_mim][PF_6_]/[C_4_mim]Cl equimolar mixture, intermediate values for almost all the solutes (considering the $K_{L}$ values of the pure ILs) were observed, and for the polar solutes (alcohols and water), the $K_{L}$ values are closer to [C_4_mim]Cl than to [C_4_mim][PF_6_], as observed for the $\gamma_{13}^{\infty }$.

### 2.4. Infinite Dilution Thermodynamic Functions

The affinity between the solutes and ILs can be further explored by calculating the partial molar functions at infinite dilution, namely the Gibbs energy, ${\bar{G}}_{m}^{E,\infty }$, enthalpy, ${\bar{H}}_{m}^{E,\infty }$, and entropy, ${\bar{S}}_{m}^{E,\infty }$. These thermodynamic properties were obtained from the $\gamma_{13}^{\infty }$ data at different temperatures, using Equations (S6)–(S8), and are listed in Table S8 and displayed in Figures S4–S6.

Concerning the results for pure [C_4_mim]Cl (Figure S4A), all ${\bar{G}}_{m}^{E,\infty }$ are positive except those of the alcohols and water. The enthalpic effects were dominant over the entropic on the solvation of alkanes, cycloalkanes, alcohols, and water. For the remaining solutes, an opposite behavior was observed (higher $T_{ref}{\bar{S}}_{m}^{E,\infty }$. absolute values). In the case of systems involving [C_4_mim][PF_6_] (Figure S4C) in low polar solutes, such as alkanes and cycloalkanes, ${\bar{H}}_{m}^{E,\infty }$ is always positive, indicating that solute–IL interactions increased with temperature. For aromatic hydrocarbons and acetates, the ${\bar{H}}_{m}^{E,\infty }$ values are nearly zero, and the entropic factor predominates. For the protic solutes (alcohols and water), the excess partial molar properties are all positive in [C_4_mim][PF_6_], which results in a distribution in region (II), i.e., no affinity between ILs and those solutes that may lead to phase separation. The results of the equimolar [C_4_mim][PF_6_]/[C_4_mim]Cl mixture show different trends (Figure S4B). For almost all of the polar solutes studied, ${\bar{H}}_{m}^{E,\infty }$ and ${\bar{G}}_{m}^{E,\infty }$ are negative or close to zero, indicating their affinity with the IL mixture. Additionally, for the nonpolar solutes, the enthalpic effect dominates over the entropic. The positive values of ${\bar{G}}_{m}^{E,\infty }$. and the negative values of $T_{ref}{\bar{S}}_{m}^{E,\infty }$ in low/medium apolar probes also indicate that their solvation in the investigated ILs is highly unfavorable. Concerning the aromatic hydrocarbons, the behavior was close to [C_4_mim][PF_6_]. 

For terpenes and terpenoids (Figures S5 and S6), $\gamma_{13}^{\infty }$ are always positive; therefore, the thermodynamic energies fall in regions (II) and (III) where entropic contributions are usually dominant. Considering the nonpolar hydrocarbons terpenes, the ${\bar{G}}_{m}^{E,\infty }$ and ${\bar{H}}_{m}^{E,\infty }$ are positive while the $T_{ref}{\bar{S}}_{m}^{E,\infty }$ are negative in all the IL systems, which indicates that their solvation in these ILs is highly unfavorable. The increase of the entropic contribution when replacing the IL anion [PF_6_]^−^ for the polar chloride is clear for polar terpenoids.

### 2.5. Separation Factors

To assess the potentialities of the ILs as separation agents of important separation problems, the selectivities ($S_{ij}^{\infty }$), capacities ($k_{j}^{\infty }$), and solvent performance indices ($Q_{ij}^{\infty }$) were calculated using the $\gamma_{13}^{\infty }$ data, using Equations (S10)–(S12), respectively. The results for pairs of terpenes or phenolic terpenoids representative of essential oils are presented in Tables S9 and S10, respectively. Table S11 reports the results for common separations important in fuel processing. Data for other ILs available in the literature are also included.

For a solvent to be considered suitable for a given separation, it should present both high selectivity and capacity and, therefore, a high performance index. Low $S_{ij}^{\infty }$ and $k_{j}^{\infty }$ mean poor separation efficiencies and poor solute–IL affinities, respectively, thus demanding large amounts of solvent [47,48].

#### 2.5.1. Terpenes Fractionation 

**Figure 3:**
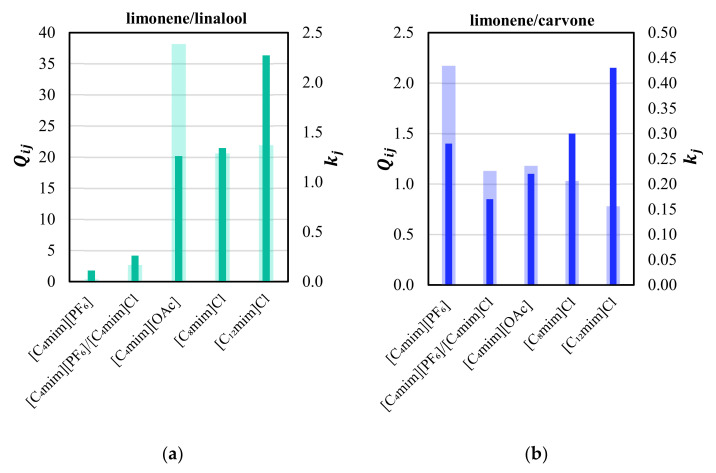
Comparison between the solvent performance indices, $Q_{ij}^{\infty }$, (light colored bars) and capacities, $k_{j}^{\infty }$, (dark colored bars) at infinite dilution for the separation of limonene/linalool (**a**) and limonene/carvone (**b**) at 403.2 K in [C_4_mim][PF_6_], equimolar [C_4_mim][PF_6_]/[C_4_mim]Cl mixture, [C_4_mim][OAc] [11], [C_8_mim]Cl [47], and [C_12_mim]Cl [47].

Hydrocarbon terpenes are known to act as impurities in some essential oils due to their low solubility in aqueous and alcoholic solutions, and due to the fact that these nonpolar fractions are more prone to oxidation processes. They can produce off-flavors that ultimately deteriorate the quality of the marketed EOs [49,50,51]. Thus, their removal (deterpenation) aims to separate the hydrocarbons from the oxygenated compounds, which are highly odoriferous and flavored [33]. In this scenario, the fractionation of the pair limonene/linalool, found in citrus EO [52,53], has been deeply investigated in the literature using ILs [11,12,16,18,25,34,35,54,55], due to the importance of this particular oil. Figure 3 shows that the highest capacity of linalool is observed in [C_12_mim]Cl, favored by the increase in the cation alkyl chain length. The pair limonene/carvone is representative of spearmint and caraway EOs, reaching up to 90% of the oil [56,57,58,59,60,61]. As shown in Figure 3, the best results for their separation were obtained with [C_4_mim][PF_6_].

Oregano EO is mainly composed of carvacrol and/or thymol (depending on the plant origin) [62,63], being responsible for the oil’s antioxidant effects [64,65,66]. In this case, it is important to perform the separation of these major constituents from other hydrocarbon terpenes to increase the oregano EO value. Table S10 shows an overview of the separation factors of selected mixtures involving phenolic terpenoids in [C_4_mim][PF_6_] and [C_4_mim][CF_3_SO_3_] for which the experimental data are also available in the literature [25]. Overall, $Q_{ij}^{\infty }$ are low, namely because of the low capacities observed. Comparing the experimental results of [C_4_mim][PF_6_] with the data reported by Martins et al. [25], higher $Q_{ij}^{\infty }$ values were observed for [C_4_mim][CF_3_SO_3_] for all the phenolic terpenoid/hydrocarbon terpene separation problems investigated. This is an interesting point, showing cases where, maintaining the same cation ([C_4_mim]^+^), the combination of two ILs with anions presenting very different polarities cannot approach the performance using an intermediate polar anion [67] only. Globally, for all the other terpene separations usually discussed in our works [11,12,16,25,35,47], the combination of [C_4_mim][PF_6_] and [C_4_mim][Cl] does not give better results than those already published.

#### 2.5.2. Fuel Processing 

In fuel processing technologies, solvents are of the utmost importance in different stages of the process. Thus, engineers are constantly looking for more efficient and environmentally friendly processes [68,69]. The separation of aromatic compounds from C_4_–C_10_ aliphatic hydrocarbons and the removal of nitrogen/sulphur compounds from fossil fuels is crucial to improving the final product quality and decreasing the ecological damage to the environment and human health. 

**Figure 4:**
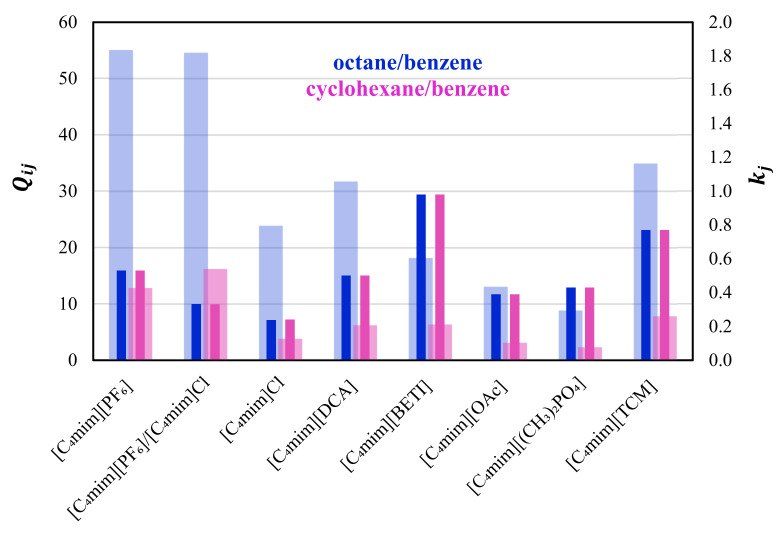
Comparison between the solvent performance index, $Q_{ij}^{\infty }$, (light colored bars) and capacities, $k_{j}^{\infty }$, (dark colored bars) at infinite dilution for the separation of octane/benzene (purple) and cyclohexane/benzene (pink) at 333.2 K in [C_4_mim][PF_6_], equimolar [C_4_mim][PF_6_]/[C_4_mim]Cl mixture, [C_4_mim]Cl [16], [C_4_mim][DCA] [17], [C_4_mim][BETI] [41], [C_4_mim][OAc] [11], [C_4_mim][(CH_3_)_2_PO_4_] [16], and [C_4_mim][TCM] [42].

Removal of aromatic impurities from fuels: The removal of aromatics from aliphatic compounds is one of the most challenging issues in refinery processes, since these compounds have close boiling points and several combinations form azeotropes [23,70]. The separations are typically performed by employing conventional processes, such as liquid–liquid extraction and extractive or azeotropic distillation using polar solvents, such as sulfolane, N-formyl morpholine (NFM), and N-methyl pyrrolidone (NMP) [23,47]. However, these organic solvents have severe drawbacks such as high volatilities, which implies extractant loss, high regeneration costs, and higher toxicity for humans and the environment [23,71]. ILs are interesting alternatives, mainly because of the low vapor pressure that allows an easy solvent recovery by flash stripping or distillation [10]. Thus, this extraction using ILs is expected to require fewer steps and energy consumption than conventional organic solvents. Inhere, two pairs of common aromatic/aliphatic separation processes in the petrochemical industry (octane/benzene and cyclohexane/benzene) are investigated. Results for the separation factors are presented in Figure 4 and listed in Table S11, along with data from the literature [11,16,17,21,27,41,42,72,73].

As shown in Figure 4, the highest solvent performance index is observed for the separation of octane and benzene using pure [C_4_mim][PF_6_] ($Q_{octane/benzene}^{\infty \;}$ = 55.02), followed by its equimolar mixture with [C_4_mim]Cl. The $k_{j}^{\infty }$ value is slightly higher in [C_4_mim][PF_6_] than in the equimolar mixture, however, both are lower than the value reported with [C_4_mim][BETI] [41]. Analyzing the results for the cyclohexane/benzene pair, separation factors are, in general, low, with the equimolar mixture showing the highest solvent performance index value (16.17) among all the methylimidazolium-based ILs previously studied [11,16,17,21,27,41,42,72,73]. Making an overview of the results for all methylimidazolium-based ILs present in Table S11 and considering the previous information about the Kamlet–Taft β parameter [43,44] for the different anions, it seems that more polar anions tend to present lower solvent performance indexes (due to the low capacities) for the aromatic/aliphatic separation problems. 

Desulfurization and denitrification of fuels: Fuels are complex, multicomponent mixtures of saturated, unsaturated, and aromatic hydrocarbons, including sulfur and aromatic nitrogen compounds that contribute to environmental pollution and human respiratory diseases. Additionally, they are known for inhibiting the hydrodesulfurization process, which is undesirable in petroleum refining processes [47,74], and can be responsible for potential equipment corrosion [47]. However, these compounds are difficult to remove because of their high molecular weight and boiling point. 

The use of ILs is promising for desulfurization and denitrification processes once they have the ability of extracting aromatic sulfur- and nitrogen-containing compounds at ambient conditions, and they can be tailored to present low affinity with the aliphatic hydrocarbons present in the fuels [11,19]. The extractive desulfurization with ILs was proved to have more potential than the traditional hydrodesulfurization technology in the removal of thiophenic sulfur compounds [75]. 

**Figure 5:**
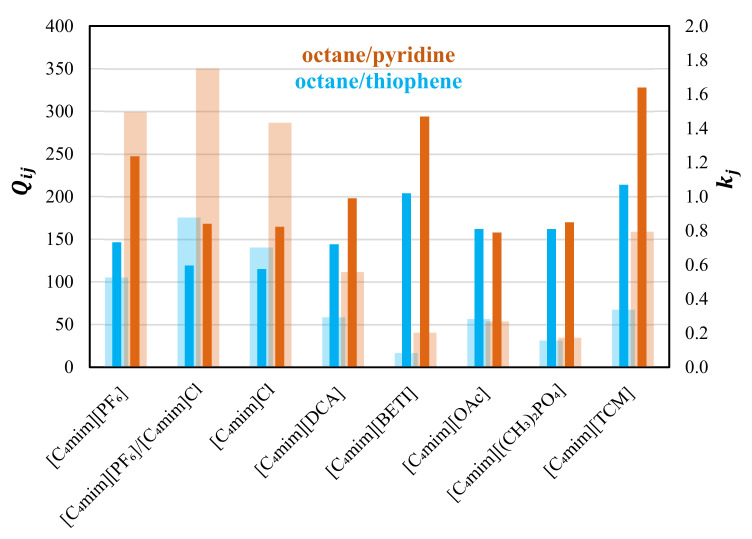
Comparison between the solvent performance index, $Q_{ij}^{\infty }$, (light colored bars) and capacities, $k_{j}$, (dark colored bars) at infinite dilution for the separation of octane/thiophene (blue) and octane/pyridine (orange) at 333.2 K in [C_4_mim][PF_6_], equimolar [C_4_mim][PF_6_]/[C_4_mim]Cl mixture, [C_4_mim]Cl [16], [C_4_mim][DCA] [17], [C_4_mim][BETI] [41], [C_4_mim][OAc] [11], [C_4_mim][(CH_3_)_2_PO_4_] [16], and [C_4_mim][TCM] [42].

This research focuses on the evaluation of methylimidazolium-based ILs for the separation of octane/pyridine and octane/thiophene (Table S11). As depicted in Figure 5, the results obtained are very promising with the three solvents inhere evaluated showing $Q_{ij}^{\infty }$ over 100. For the pair octane/pyridine, the best results were obtained with the equimolar mixture ($Q_{octane/pyridine}^{\infty \;}$ = 350), followed by pure [C_4_mim][PF_6_]. Regarding capacities, a very interesting compromise is observed in [C_4_mim][PF_6_] where $k_{pyridine}^{\infty }$ = 1.24. For the separation of the octane/thiophene mixture, similar capacities are observed in [C_4_mim][PF_6_], [C_4_mim]Cl, and their mixture. However, the latter presents the highest solvent performance index (177).

In desulfurization and denitrification processes, as well as for the separations of aromatic hydrocarbons from the aliphatic ones, the results show that a combination of the anions chloride and hexafluorophosphate results in higher selectivities and intermediate capacities when compared with the pure ILs. Additionally, the results herein obtained for the equimolar mixture stand out when compared with the eutectic mixture of choline and glycerol [31] (Table S12). Compared to the other ILs displayed in Table S11, the solvents studied here are promising, showing their potential to carry out the removal of contaminants from fuels and the removal of aromatic from aliphatic hydrocarbons.

## 3. Materials and Methods

The experimental procedure—including the column packing and the chromatographic methodology—for the measurement of the activity coefficient at infinite dilution is detailed in our previous works [11,16,25] and summarized in Section S1 of the SI. Details of the investigated ILs are presented in Table S1. The chemical structure, supplier, boiling temperature, and purity of the organic solutes are shown in Table S2. Solute stereochemistry is omitted in the manuscript. To reduce water and volatile impurities, ILs were dried before use (vacuum = 0.1 Pa, continuous stirring, room temperature, >48 h). The thermodynamic background for the calculation of the activity coefficients at infinite dilution, molar excess thermodynamic functions, and separation factors (selectivity, capacity, and solvent performance indices) are displayed in Section S2 of the SI.

## 4. Conclusions

This work studies methylimidazolium-based ILs as alternative solvents for some important industrial processes, such as terpene fractionation and fuel processing. In general, the capacities of the nonpolar and polar aprotic solutes are higher in the more apolar [C_4_mim][PF_6_] and the lowest in [C_4_mim]Cl, with intermediate values for the equimolar IL mixture. For alcohols and water, the opposite trend was observed. The results obtained show the studied ILs are among the solvents having the best performance indices compared to other methylimidazolium-based ILs for the separation of several model mixtures (octane/benzene, cyclohexane/benzene, octane/thiophene, and octane/pyridine). More particularly, the results suggest that the equimolar mixture ([C_4_mim][PF_6_]/[C_4_mim]Cl) is a suitable solvent for the desulfurization and denitrification of fuels. The IL mixture showed the best solvent performance indices among the corresponding organic solvents and previously investigated ILs. Regarding terpenes, the best affinities were observed with pure [C_4_mim][PF_6_], followed by the equimolar mixture and finally [C_4_mim]Cl, but none of these ILs present separation factors that turn separation efficiencies among terpenes higher. Globally, this study shows the importance and potential of mixing ionic liquids with different cations and/or anions, tailoring their polarities for specific separation problems involving common organic solutes and terpenes. 

The activity coefficients at infinite dilution and the derived separation properties provide valuable information for screening potential entrainers for liquid –liquid extraction or distillation processes. In future works, the application of the most promising solvents in real mixtures should be considered by studying the phase equilibria at higher IL concentrations, closer to practical industrial conditions, by experimental and modeling approaches. 

## Data Availability

Not applicable.

## Supplementary Materials

The following supporting information can be downloaded at: https://www.mdpi.com/article/10.3390/molecules28062456/s1, Section S1: Chemicals and experimental details; Section S2: Thermodynamic background; Section S3: Results and discussion on the activity coefficients at infinite dilution, gas liquid partition coefficients, limiting partial molar excess properties, and fractionation factors.
