# Supplementary material for: The Role of the Anion in Imidazolium-Based Ionic Liquids for Fuel and Terpenes Processing
Source: Molecules. 2023 Mar 7;28(6):2456. doi: 10.3390/molecules28062456 (PMC10057593; doi:10.3390/molecules28062456)
Supplement: Supplementary file 1 [file molecules-28-02456-s001.zip › molecules-2212311-supplementary.pdf]

Supporting Information

The Role of the Anion in Imidazolium-Based Ionic Liquids for Fuel and Terpenes Processing

Aline Zambom[a,b], Sérgio M. Vilas-Boas[a,b,c], Liliana P. Silva[c], Mónia A. R. Martins[a,b,c], Olga Ferreira[a,b], Simão P. Pinho[a,b,*]

[a]Centro de Investigação de Montanha (CIMO), Instituto Politécnico de Bragança, Campus de Santa Apolónia, 5300-253 Bragança, Portugal

[b]Laboratório para a Sustentabilidade e Tecnologia em Regiões de Montanha, Instituto Politécnico de Bragança, Campus de Santa Apolónia, 5300-253 Bragança, Portugal

[c]CICECO, Aveiro Institute of Materials, Complexo de Laboratórios Tecnológicos, University of Aveiro, Campus Universitário de Santiago, Aveiro, Portugal

*Corresponding author: Simão P. Pinho

e-mail address: spinho@ipb.pt

Phone: +351 273 303 086

Fax: +351 273 313 051

Section S1 – Chemicals and experimental details

Table S1. ILs chemical structures and properties (melting temperature T_m, glass transition temperature T_g, mass fraction purity as declared by the supplier w, and molecular weight Mw).

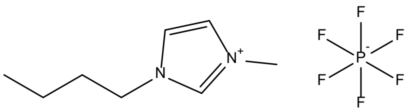	1-butyl-3-methylimidazolium hexafluorophosphate, [C_4mim][PF_6]<br>Acquired from Iolitec; w = 0.990; Mw = 284.19 g·mol^{-1}<br>T_m = 283.2 K [1]; Tg = 190.6 K [1]
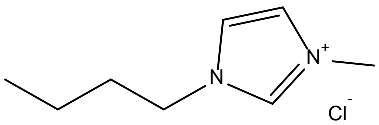	1-butyl-3-methylimidazolium chloride, [C_4mim]Cl<br>Acquired from Iolitec; w = 0.990; Mw = 174.67 g·mol-1<br>T_m = 341.95 K [2]; Tg = 197.35 K [2]

Table S2. Name, structure, source, boiling temperature, and mass fraction purity (as declared by the supplier) of the organic solutes used. Names in parentheses correspond to synonyms used in the text. Solutes stereochemistry is omitted in the manuscript.

Family	Name	Chemical structure	Supplier	Boiling temperature (K)	Purity (mass fraction)
	Water	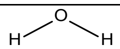	-[a]	373.15[b]	-[a]
Alkanes	Heptane	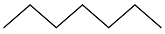	Aldrich	371.15[b]	≥ 0.990
	Octane	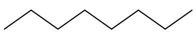	Aldrich	398.77[b]	≥ 0.990
	Nonane	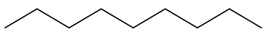	Aldrich	423.91[b]	≥ 0.990
	Decane	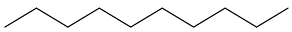	Aldrich	447.20[b]	≥ 0.990
Cycloalkanes	Cyclohexane	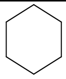	Aldrich	353.90[b]	≥ 0.990
	Methylcyclohexane	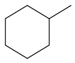	Aldrich	374.00[b]	≥ 0.990
Ketones	Propanone (Acetone)	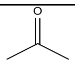	Aldrich	329.30[b]	≥ 0.999
	2-Butanone	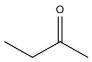	Aldrich	353.00[b]	≥ 0.990
Ether	Ethoxyethane (Diethyl ether)	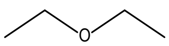	Aldrich	307.70[b]	≥ 0.999
Cyclic Ethers	Oxolane (THF)	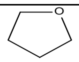	Aldrich	339.00[b]	≥ 0.999
	1,4-dioxane	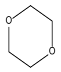	Aldrich	374.30[b]	≥ 0.998
Aromatic Hydrocarbons	Benzene	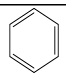	Aldrich	353.22[b]	≥ 0.998
	Toluene	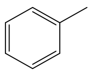	Aldrich	383.75[b]	≥ 0.998
	Ethylbenzene	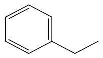	Aldrich	409.35[b]	≥ 0.998

	p-xylene	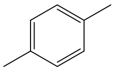	Aldrich	411.5[b]	≥ 0.990
Esters	Methyl acetate	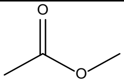	Aldrich	330.00[b]	≥ 0.998
	Vinyl acetate	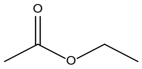	Riedel-de-Häen	345.70[b]	≥ 0.990
	Ethyl acetate	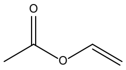	Aldrich	350.20[b]	≥ 0.998
Alcohols	Methanol	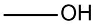	Aldrich	337.80[b]	≥ 0.998
	Ethanol	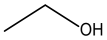	Aldrich	351.50[b]	≥ 0.999
	1-propanol	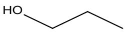	Aldrich	370.30[b]	≥ 0.999
	2-propanol	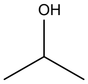	Fluka	355.50[b]	≥ 0.995
	2-methyl-1-propanol (Isobutanol)	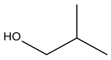	Aldrich	380.80[b]	≥ 0.998
	1-butanol	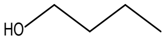	Aldrich	390.60[b]	≥ 0.995
	2-butanol	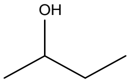	Aldrich	372.00[b]	≥ 0.997
	2-methyl-2-propanol (*tert*-butanol)	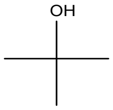	Aldrich	355.50[b]	≥ 0.999
	Acetonitrile	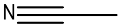	Fluka	355.15[b]	≥ 0.998
	Pyridine	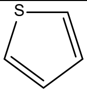	Aldrich	388.15[b]	≥ 0.990
	Thiophene	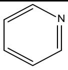	Aldrich	357.15[b]	≥ 0.980
Terpenes	α-pinene	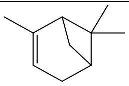	Aldrich	430.00[b]	≥ 0.990
	β-pinene	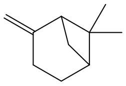	Aldrich	439.20[b]	≥ 0.970
	R-(+)-limonene	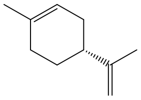	Aldrich	449.65[b]	≥ 0.990
	p-cymene	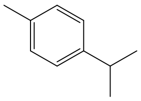	Aldrich	450.28[b]	≥ 0.990
	Myrcene	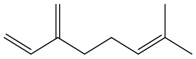	Aldrich	440.20[b]	≥ 0.990
	γ-terpinene	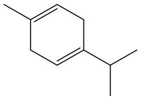	Aldrich	455.15[b]	≥ 0.970
Terpenoids	(−)-menthone	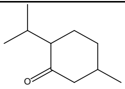	Fluka	490.79[b]	≥ 0.980

(1*R*)-(−)-fenchone	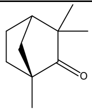	Aldrich	466.65[c]	≥ 0.970
α-pinene oxide	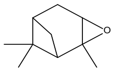	Aldrich	447.15[c]	≥ 0.990
Eucalyptol	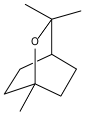	Aldrich	449.55[c]	≥ 0.970
Linalool	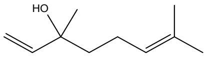	Aldrich	471.65[c]	≥ 0.980
Geraniol	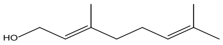	Aldrich	502.15[c]	≈ 0.950
DL-citronellol	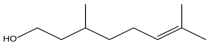	Aldrich	497.65[c]	≥ 0.980
(1*R*)-(+)-camphor	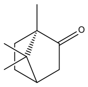	Aldrich	480.55[c]	≥ 0.980
(*S*)-(+)-carvone	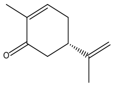	Merck	503.65[c]	≥ 0.997
L(−)-menthol	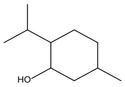	Acros	488.55[c]	≥ 0.980
(−)-isopulegol	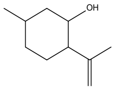	SAFC	470.15[c]	≥ 0.990
(−)-borneol	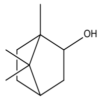	Fluka	485.15[c]	≥ 0.990
Citronellal	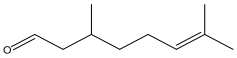	Aldrich	480.15[c]	≥ 0.950
Eugenol	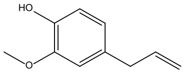	Aldrich	526.35[b]	0.990
Carvacrol	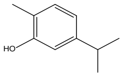	SAFC	510.15[b]	0.990
Thymol	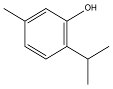	Sigma	505.65[b]	≥ 0.995

[a]Ultrapure water was used (resistivity of 18.2MΩ·cm, free particles ≥ 0.22 μm and total organic carbon < 5 μg·dm^{-3}).
[b]The boiling temperature was obtained from Yaws [3].
[c]The boiling temperature was obtained from ChemSpider [4].

Column packing and Chromatographic methodology

The column packing method follows our previous works [5–7]: the ionic liquid (IL) and the solid support (Chromosorb W/AW – DMCS, Grace, 100-120 mesh) were diluted in methanol that was then removed through vacuum-assisted rotary evaporation. The resulting solid mixture (stationary phase 45-50% in mass) was packed into an in-house glass column (length: 1 m;

internal diameter: 0.4 cm) using a vacuum pump. Before measurements, the column was pre-conditioned, *i.e.*, a stream of helium gas (carrier gas) was passed through for at least 6 h at 393.2 K to facilitate the removal of eventual impurities. The solute retention time in each IL was then measured by a Varian CP-3380 gas chromatograph equipped with a 1041 on-column injector and a thermal conductivity detector (TCD). A Swagelok S model pressure transducer with an accuracy of 0.25% BFSL was used to measure the column inlet pressure. The outlet temperature (±0.1 K), the atmospheric pressure (±0.05 atm), and the outlet flow rate (relative uncertainty of 6%) were measured with a precision gas flowmeter (Agilent, model 5067-0223). During the analyses with traditional organic solvents and water, the injector and detector temperatures were set at 503.2 K and 523.2 K, respectively. For the analyses involving terpenes and terpenoids, the injector and detector temperatures were set to 553.2 K and 573.2 K, respectively. To achieve infinite dilution conditions, the solutes were injected in the column in the volume range of (0.2-0.5) μL. Together with the solute, air was injected as a non-retained component. Retention times were calculated by the difference of the retention times of the solute, t_R, and air, t_G. The experiments were performed at least at three different temperatures in the range 333.2-453.2 K and each experiment was repeated at least twice. For a set of solutes (minimum 10 solutes, for each ionic liquid in the stationary phase, including compounds from different families), the retention times were measured in two independent columns at three temperatures. The γ_{13}^{∞} values of the two independent columns have a repeatability with a global coefficient of variation (mean value) of 3.25%. An XP205 Mettler Toledo scale (readability ± 0.01 mg) was used to prepare the equimolar [C_4mim][PF_6]/[C_4mim]Cl mixture (0.501:0.499 mole ratio).

Section S2 - Thermodynamic background

Activity Coefficient at Infinite Dilution

The retention times obtained by the inverse gas chromatography (IGC) methodology were used to calculate the activity coefficients at infinite dilution, γ_{13}^{∞}, for a solute (1) partitioning between a carrier gas (2) and a non-volatile liquid solvent (3), using the methodologies developed by Everett [8] and Cruickshank et al. [9] in the 1960s, as presented in **Eq. (S1)**:

$$\ln \gamma_{13}^{\infty} = \ln \frac{n_3 RT}{V_N p_1^*} - \frac{p_1^*(B_{11} - V_1^*)}{RT} + \frac{p_0 J_2^3 (2B_{12} - V_1^{\infty})}{RT} \qquad \textbf{(S1)}$$

n_3	number of moles of solvent packed in the column
R	ideal gas constant
T	absolute temperature of the column (regulated by the GC oven)
V_N	net retention volume of the solute - **Eq. (S2)**
p_1^*	saturated vapor pressure of the solute at the column temperature
B_{11}	second virial coefficient of the pure solute
V_1^*	molar volume of the solute
p_0	column outlet pressure
J_2^3	pressure correction factor - **Eq. (S3)**
B_{12}	crossed second virial coefficient of the solute and the carrier gas (helium)
V_1^{∞}	partial molar volume of the solute at infinite dilution in the solvent

V_N and J_2^3 are given by:

$$V_N = (J_2^3)^{-1} U_0 (t_R - t_G) \qquad \textbf{(S2)}$$

U_0	outlet volumetric flow rate (at the column temperature)
t_r	retention times of the solute
t_g	retention times of air (non-retained substance)

$$J_2^3 = \frac{2\left({p_i}/{p_0}\right)^3 - 1}{3\left({p_i}/{p_0}\right)^2 - 1} \qquad \textbf{(S3)}$$

p_i	column inlet pressure

As the flow rate, *U*, is measured with a flowmeter placed after the carrier gas leaves the detector, it needs to be corrected:

$$U_0 = U\,\frac{p_f}{p_0}\frac{T}{T_f} \tag{S4}$$

U_f	volumetric flow measured by the flowmeter after the carrier gas goes through the detector
p_f	pressure measured by the flowmeter after the carrier gas goes through the detector
T_f	temperature measured by the flowmeter after the carrier gas goes through the detector

To accurately estimate the column outlet pressure p_0, a linear regression between the pressure drop value (ΔP) and the volumetric flow rate was established at different temperatures using **Eq. (S5)**.

$$\Delta P = p_0 - p_f = A \cdot U + B \tag{S5}$$

The second order virial coefficients necessary in **Eq. (S1)** were estimated using the correlation proposed by Tsnonopoulos [10] and discussed, in detail, by Poling [11]. Additional solute properties required for γ_{13}^{∞} calculation, namely vapor pressure, density, critical properties, acentric factor, and dipole moment, were taken from previous works [5–7,12,13].

<u>*Excess Partial Molar Properties at Infinite Dilution*</u>

To further interpret the interactions and the measured γ_{13}^{∞} data, the excess partial molar properties at infinite dilution, namely excess enthalpy ($\bar{H}_m^{E,\infty}$) and entropy ($\bar{S}_m^{E,\infty}$) which are contributions to the excess Gibbs energy ($\bar{G}_m^{E,\infty}$), can be determined by using the linear dependence of γ_{13}^{∞} with temperature (van't Hoff plot [5,14]) using the following equations:

$$\ln \gamma_{13}^{\infty} = \frac{\bar{H}_m^{E,\infty}}{R}\frac{1}{T} - \frac{\bar{S}_m^{E,\infty}}{R} \tag{S6}$$

$$\bar{G}_m^{E,\infty} = RT ln\, \gamma_{13}^{\infty} \tag{S7}$$

And, at a reference temperature T_{ref}:

$$\bar{G}_m^{E,\infty} = \bar{H}_m^{E,\infty} - \bar{S}_m^{E,\infty}\, T_{ref} \tag{S8}$$

Gas-Liquid Partition Coefficient

The gas-liquid partition coefficients (K_L) are translated as the solute partitioning between the carrier gas (helium) and the stationary phase (IL) and can be determined from the IGC experiments according to [15]:

$$\ln(K_L) = \frac{c_1^3}{c_1^2} = \ln\frac{V_N \rho_3}{m_3} - \frac{p_0 J_2^3 (2B_{12} - V_1^\infty)}{RT} \quad \textbf{(S9)}$$

c	molar concentration of the solute (1)
m_3	mass of ionic liquid (3) in the column
ρ_3	density of ionic liquid (3)

Separation Factors

To evaluate the IL performance as an entrainer in a chemical separation problem, the selectivity between the solutes pair, i and j, (S_{ij}^∞), and the separation capacity, (k_j^∞), are calculated as follows:

$$S_{ij}^\infty = \frac{\gamma_{i3}^\infty}{\gamma_{j3}^\infty} \quad \textbf{(S10)}$$

$$k_j^\infty = \frac{1}{\gamma_{j3}^\infty} \quad \textbf{(S11)}$$

j	solute with the lowest activity coefficient in each separation pair
3	refers to the ionic liquid

For an ionic liquid to be a good separation agent, high selectivities and capacities are desirable. Unfortunately, these two parameters often counteract, *i.e.*, high values for selectivity are accompanied by low capacities. Thus, it is important to have a balance between these two parameters to be able to evaluate the IL efficiency. To this end, the solvent performance index (which fairly describes this balance) was used in this work [16,17]:

$$Q_{ij}^\infty = S_{ij}^\infty k_j^\infty \quad \textbf{(S12)}$$

Section S3 – Results and discussion

Activity coefficients at infinite dilution

Table S3. Activity coefficients at infinite dilution of the solutes in [C_4mim][PF_6], [C_4mim][PF_6]/[C_4mim]Cl equimolar mixture, and [C_4mim]Cl (from the literature [5,6]).[a]

Solute / *T* / K	[C_4mim][PF_6][b]						[C_4mim][PF_6]/[C_4mim]Cl equimolar mixture[c]						[C_4mim]Cl[d,e,f]					
	**333.15**	**343.15**	**353.15**	**363.15**	**373.15**	**383.15**	**333.15**	**343.15**	**353.15**	**363.15**	**373.15**	**383.15**	**333.15**	**343.15**	**353.15**	**363.15**	**373.15**	**383.15**
Heptane	154.49	137.02	123.64	117.03	101.66	88.29	-	-	-	-	-	-	-	-	-	-	-	-
Octane	195.70	178.52	167.14	151.10	133.73	124.94	495.23	435.64	406.29	328.87		-	422.72	380.73	344.95	314.23	287.68	264.59
Nonane	254.50	229.87	206.11	192.74	171.80	162.79	573.68	527.96	482.32	416.77	380.43	341.56	597.90	541.96	494.00	452.59	416.60	385.14
Decane	338.66	310.84	276.48	250.87	230.93	211.51	704.16	649.55	584.53	508.29	475.14	437.91	875.50	795.82	727.31	668.00	616.33	571.06
Cyclohexane	45.47	41.03	38.27	35.43	32.16	29.88	147.67	117.23	95.78	78.24	-	-	66.22	61.09	56.61	52.69	49.22	46.15
Methylcyclohexane	62.95	56.98	53.96	49.19	45.48	42.32	183.48	157.92	138.15	114.31	-	-	101.50	93.88	87.21	81.35	76.17	71.56
Benzene	1.89	1.91	1.93	1.96	1.98	2.01	3.01	2.99	2.96	2.92	2.91	2.88	4.21	4.33	4.44	4.55	4.66	4.77
Toluene	2.92	2.95	2.97	2.99	3.02	3.04	4.83	4.82	4.80	4.65	4.56	4.55	7.23	7.44	7.65	7.85	8.04	8.23
Ethylbenzene	5.23	5.27	5.30	5.34	5.32	5.35	8.58	8.37	8.39	8.24	8.16	8.23	12.55	12.78	13.00	13.21	13.41	13.61
p-Xylene	4.40	4.48	4.47	4.50	4.48	4.53	7.45	7.46	7.41	7.27	7.26	7.26	12.95	13.20	13.45	13.69	13.92	14.14
Methyl acetate	1.24	1.26	1.30	1.32	1.36	1.40	2.47	2.42	2.42	2.39	2.40	2.37	4.56	4.66	4.76	4.86	4.95	5.05
Ethyl acetate	1.95	1.97	2.00	2.03	2.07	-	4.20	4.13	4.05	3.99	3.91	3.85	8.88	8.94	8.99	9.04	9.09	9.13
Vinyl acetate	1.71	1.74	1.76	1.79	1.82	1.85	3.22	3.17	3.12	3.09	3.06	3.02	5.28	5.43	5.57	5.70	5.83	5.96
THF	1.52	1.54	1.57	1.59	1.62	1.65	3.00	1.26	1.35	1.41	1.43	2.77	5.62	5.64	5.65	5.66	5.68	5.69
1,4-dioxane	0.93	0.97	1.00	1.03	1.07	1.10	1.63	1.68	1.73	1.75	1.77	1.77	3.22	3.32	3.42	3.51	3.60	3.69
Diethyl ether	7.89	7.93	7.92	7.90	7.76	7.62	25.82	24.48	23.27	22.24	22.68	-	20.22	19.80	19.41	19.06	18.72	18.41
Acetonitrile	0.51	0.53	0.53	0.54	0.54	0.55	0.75	0.76	0.76	0.77	0.78	0.78	0.98	1.03	1.09	1.14	1.19	1.25
Pyridine	0.81	0.86	0.89	0.94	0.97	1.03	1.19	1.23	1.29	1.32	1.37	1.43	1.21	1.31	1.41	1.51	1.61	1.71
Thiophene	1.36	1.39	1.41	1.43	1.46	1.48	1.68	0.76	0.77	0.78	0.78	1.77	1.74	1.86	1.98	2.10	2.22	2.34
Acetone	0.71	0.74	0.75	0.77	0.79	0.82	1.42	1.43	1.44	1.45	1.45	1.47	2.85	2.93	3.01	3.08	3.15	3.22
2-Butanone	1.07	1.10	1.12	1.15	1.18	1.22	2.14	2.14	2.15	2.14	2.15	2.15	-	-	-	-	-	-
Methanol	1.93	1.83	1.77	1.67	1.57	1.53	0.21	0.23	0.24	0.26	0.27	0.27	0.09	0.10	0.11	0.12	0.13	0.14
Ethanol	2.58	2.35	2.20	1.99	1.80	1.71	0.36	0.37	0.38	0.38	0.39	0.39	0.18	0.20	0.22	0.23	0.25	0.27
1-propanol	3.47	3.14	-	2.64	2.41	2.28	0.48	0.49	0.49	0.50	0.51	0.51	0.23	0.25	0.28	0.31	0.33	0.36
2-propanol	3.18	2.88	2.65	-	2.24	2.13	0.60	0.61	0.62	0.62	0.62	0.62	0.30	0.34	0.37	0.41	0.44	0.48
Isobutanol	4.57	4.05	3.66	3.33	3.07	2.87	0.63	0.64	0.64	0.64	0.64	0.68	0.30	0.33	0.37	0.40	0.44	0.48
1-butanol	4.74	4.24	3.82	3.45	3.16	2.95	0.66	0.67	0.67	0.68	0.68	0.84	0.32	0.35	0.39	0.42	0.46	0.50
2-butanol	3.96	3.62	3.31	3.05	2.88	2.73	0.79	0.81	0.82	0.83	0.84	0.64	0.41	0.45	0.50	0.55	0.61	0.66
Tert-butanol	3.73	3.45	3.24	3.08	2.94	2.82	1.03	1.06	1.09	1.11	1.12	1.12	0.49	0.55	0.62	0.70	0.77	0.85
Water	2.83	2.52	2.21	2.00	1.82	1.63	0.13	0.15	0.16	0.16		0.17	0.03	0.04	0.04	0.05	0.05	0.06

**Terpenes/terpenoids**	**353.15**	**363.15**	**373.15**	**383.15**	**393.15**	**403.15**	**353.15**	**363.15**	**373.15**	**383.15**	**393.15**	**403.15**	**353.15**	**363.15**	**373.15**	**383.15**	**393.15**	**403.15**
α-pinene	54.62	51.95	48.97	46.92	44.11	42.70	101.58	78.66	75.24	74.81	73.37	64.28	414.66	383.21	355.64	331.35	309.83	290.67
β-pinene	34.09	32.72	31.36	30.78	29.21	28.67	59.82	51.01	49.25	44.38	41.78	37.97	256.84	244.63	233.60	223.61	214.52	206.23
Limonene	33.66	31.22	30.49	29.38	28.20	27.55	53.70	48.78	48.67	43.66	39.20	38.83	-	-	-	-	-	-
Myrcene	31.05	30.74	30.24	29.90	29.67	29.57	51.16	51.19	46.09	46.38	44.03	42.77	-	-	-	-	-	-
γ-terpinene	24.67	24.42	24.11	23.85	23.79	23.55	38.04	37.16	35.64	34.90	35.01	34.62	-	-	-	-	-	-
p-cymene	13.02	12.93	12.90	12.64	12.58	12.52	22.73	21.52	20.50	21.24	20.27	20.20	-	-	-	-	-	-
Eucalyptol	17.92	17.31	17.19	16.14	15.86	15.67	35.83	32.67	29.57	30.46	28.02	26.30	189.63	182.98	176.90	171.33	166.21	161.48
**Low volatile terpenoids**	**383.15**	**393.15**	**403.15**	**413.15**	**423.15**	**433.15**	**383.15**	**393.15**	**403.15**	**413.15**	**423.15**	**433.15**	**383.15**	**393.15**	**403.15**	**413.15**	**423.15**	**433.15**
α-pinene oxide	6.25	6.51	6.67	6.83	7.07	7.21	11.55	11.69	11.94	11.99	12.12	12.92	79.52	83.01	86.45	89.86	93.23	96.55
Fenchone	5.62	5.66	5.72	5.73	5.73	5.76	10.87	10.91	11.31	10.73	10.78	11.08	67.88	66.08	64.41	62.87	61.43	60.10
Menthone	6.80	6.84	6.92	7.00	7.04	7.05	13.21	13.26	13.26	13.08	12.88	12.64	92.79	92.29	91.82	91.37	90.95	90.55
Carvone	3.41	3.49	3.57	3.68	3.75	3.85	5.64	5.65	5.86	5.90	6.13	6.40	35.70	36.16	36.62	37.05	37.47	37.87
Isopulegol	7.02	6.92	6.81	6.73	6.54	6.48	3.02	3.25	3.34	3.48	3.75	3.94	6.78	7.24	7.70	8.17	8.64	9.11
Citronellol	11.11	10.64	-	9.31	8.76	8.35	2.83	2.90	3.01	3.03	3.05	3.10	6.29	6.77	7.27	7.77	8.28	8.79
Geraniol	8.77	8.15	7.80	7.45	7.00	6.73	-	2.16	2.20	2.30	2.39	2.50	4.15	4.60	5.08	5.57	6.09	6.62
Linalool	8.23	7.98	7.96	7.89	7.88	8.01	2.86	3.05	3.31	3.49	3.67	4.15	36.17	51.90	73.11	101.26	138.05	185.47
Citronellal	8.50	8.28	8.14	8.04	7.87	7.67	15.25	14.42	13.83	12.33	12.24	-	-	-	-	-	-	-
Camphor	4.38	4.43	4.54	4.64	4.73	4.85	-	8.45	8.47	8.39	8.74	8.91	51.23	51.30	51.36	51.42	51.48	51.54
Borneol	10.97	10.02	9.09	8.15	7.51	7.00	2.91	2.90	2.80	2.72	2.73	-	4.78	4.28	3.85	3.48	3.17	2.89
Menthol	11.54	10.97	10.71	10.25	9.92	9.72	3.81	3.98	4.13	4.19	4.46	4.71	8.23	8.84	9.46	10.10	10.74	11.39
**Phenolic Terpenoids**	**413.15**	**423.15**	**433.15**	**443.15**	**453.15**													
Eugenol	3.21	3.46	3.62	3.90	4.12													
Carvacrol	1.80	1.92	2.05	2.17	2.30													
Thymol	1.84	1.92	2.01	2.09	2.13													

[a]The estimated uncertainties in pressure, temperature, and γ_{13}^{∞} are $u(T) = 0.1$ K, $u_r(p) = 0.05p$, and $u(\gamma_{13}^{\infty}) = 0.04$.

[b]Colunm Packing: 50% of IL, $n_3 = 9.48$ mmol.

[c]Colunm Packing: 50% of IL, $n_3 = 10.75$ mmol.

[d]Colunm Packing: 51.1% of IL, $n_3 = 17.02$ mmol.

[e]The γ_{13}^{∞} of water and organic solutes in [C$_4$mim]Cl were extrapolated at 333.15-353.15 K and interpolated at 363.15-383.15 K using data obtained in a temperature range of 358.15-388.15 K reported by Martins et al. [5].

[f]The γ_{13}^{∞} of terpenes and terpenoids in [C$_4$mim]Cl were extrapolated at 353.15-393.15 K and interpolated at 403.15-433.15 K using data obtained in a temperature range of 398.15-448.25 K reported by Martins et al. [6].

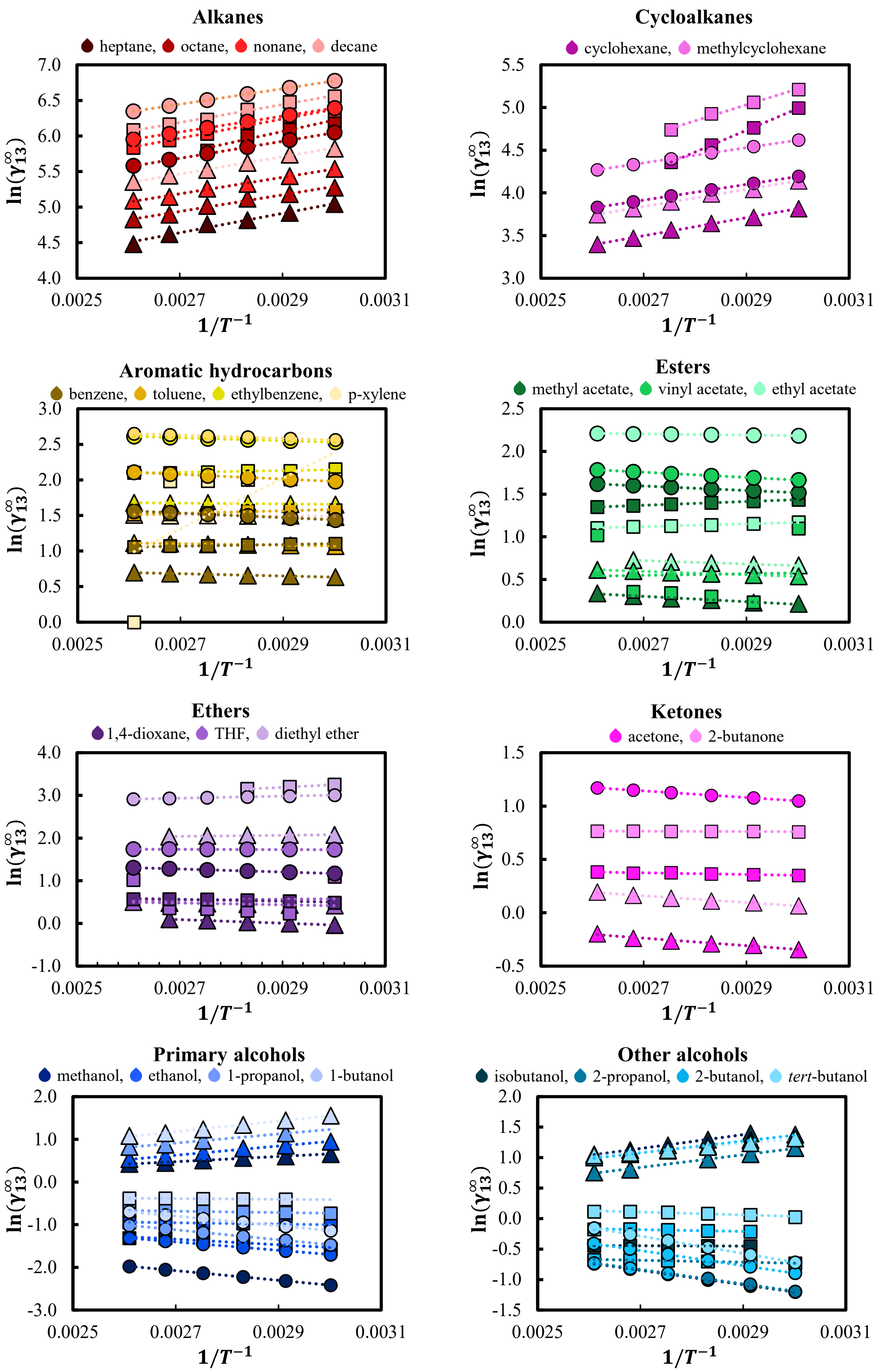
Alkanes
heptane, octane, nonane, decane
Cycloalkanes
cyclohexane, methylcyclohexane
Aromatic hydrocarbons
benzene, toluene, ethylbenzene, p-xylene
Esters
methyl acetate, vinyl acetate, ethyl acetate
Ethers
1,4-dioxane, THF, diethyl ether
Ketones
acetone, 2-butanone
Primary alcohols
methanol, ethanol, 1-propanol, 1-butanol
Other alcohols
isobutanol, 2-propanol, 2-butanol, tert-butanol
$\ln(\gamma_{13}^{\infty})$
$1/T^{-1}$
0.0025
0.0027
0.0029
0.0031

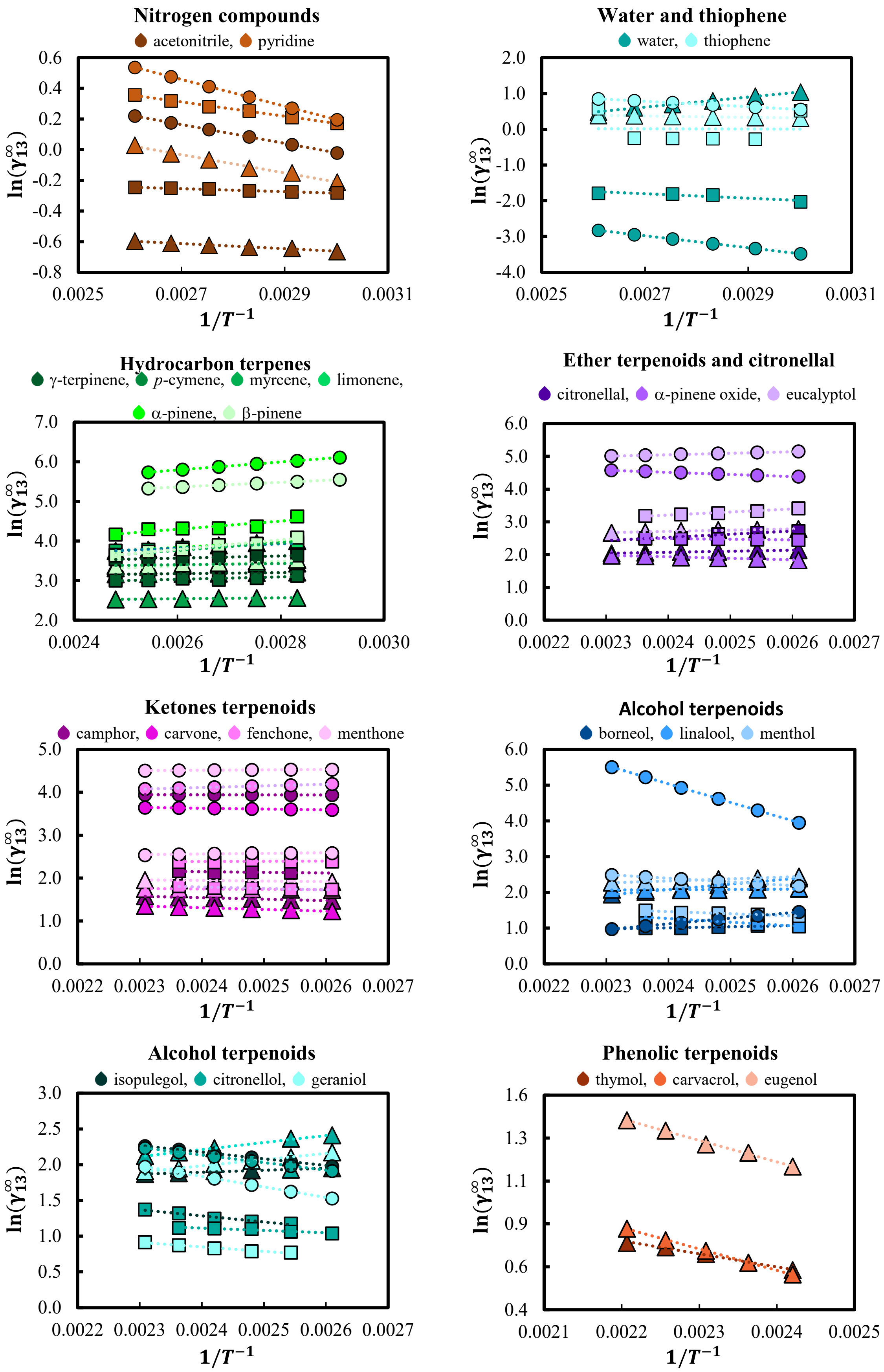

Figure S1. Representation of ln γ_{13}^{∞} as function of $1/T$ (K^{-1}) of the solutes in (●), [C_4mim]Cl [5,6]; (■), [C_4mim][PF_6]/[C_4mim]Cl mixture; and (▲), [C_4mim][PF_6].

Comparison with literature data

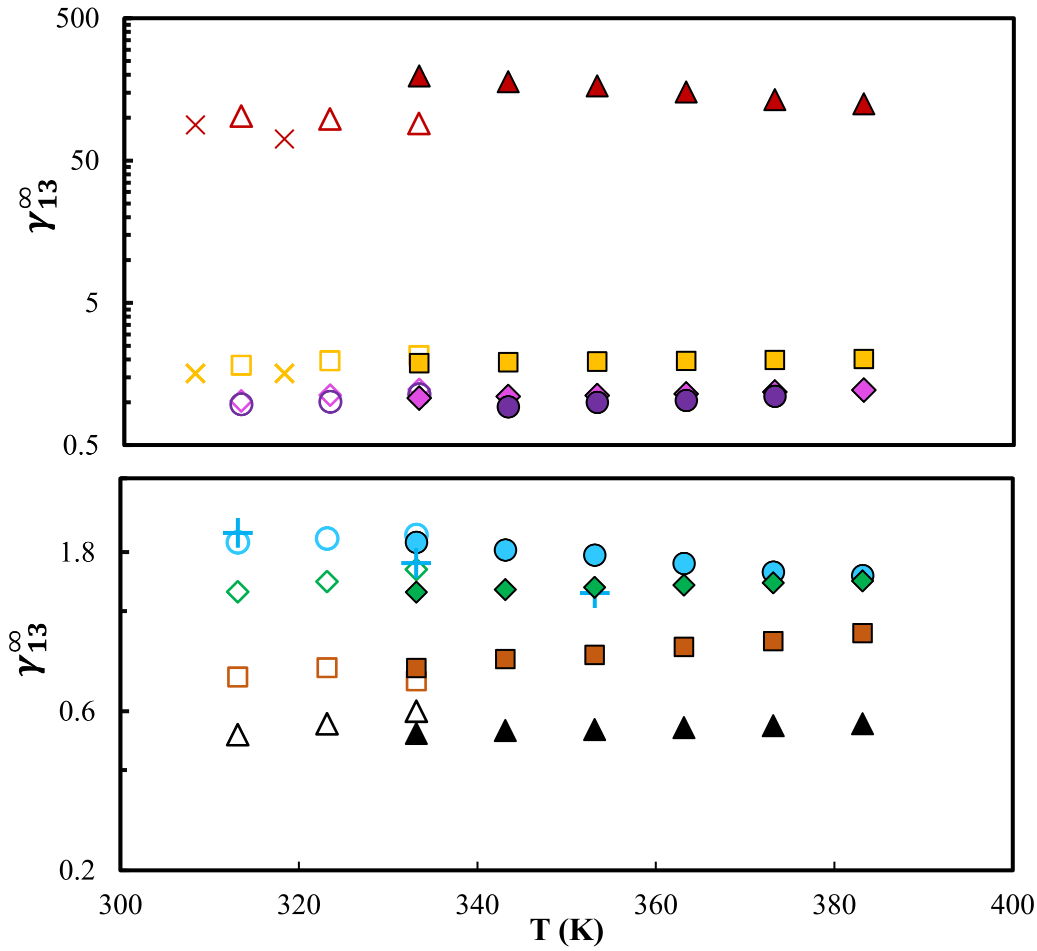

Figure S2. Comparison of the experimental activity coefficients at infinite dilution with values from the literature for [C_4mim][PF_6]. Colour code: ▲ octane, ■ benzene, ● 1,4-dioxane, ◆ 2-butanone, ● methanol, ■ pyridine, ▲ acetonitrile, and ◆ thiophene. Full symbols correspond to experimental data measured in this work, and empty symbols correspond to literature data from Mutelet et al. [18]; (✕), Zhu et al. [19]; and (+), Dobryakov et al. [20].

Solvatochromic parameters

Table S4. Solvatochromic parameters, namely dipolarity/polarizability (π^*), hydrogen bond acidity (α), and hydrogen bond basicity (β) for some methylimidazolium-based ILs [21,22].

Ionic Liquid	π^*	α	β	Source
1-butyl-3-methylimidazolium bis(trifluoromethylsulfonyl)imide, [C_4mim][BETI]	0.83	0.55	0.42	[21]
1-butyl-3-methylimidazolium hexafluorophosphate, [C_4mim][PF_6]	0.90	0.54	0.44	[21]
1-butyl-3-methylimidazolium tricyanomethanide, [C_4mim][TCM]	0.94	0.51	0.54	[21]
1-butyl-3-methylimidazolium dicyanamide, [C_4mim][DCA]	0.98	0.44	0.64	[21]
1-butyl-3-methylimidazolium chloride, [C_4mim]Cl	1.13	0.32	0.95	[21]
1-butyl-3-methylimidazolium dimethylphosphate, [C_4mim][$(CH_3)_2PO_4$]	0.98	0.45	1.13	[22]
1-butyl-3-methyl-imidazolium acetate, [C_4mim][OAc]	0.89	0.57	1.18	[21]

Density measurements

Density of pure [C_4mim][PF_6] was calculated as an average of available literature data reported in the literature [23–25], **Table S5**. The density of the equimolar [C_4mim][PF_6]/[C_4mim]Cl mixture (**Table S6**) was estimated assuming an ideal mixture, following the methodology proposed by Rebelo et al. [26] and previously employed by us [7,12]. For that, the density data available in the literature of pure [C_4mim]Cl [5,27–35] and [C_4mim][PF_6] [23–25] (**Table S5**) were used.

Table S5. Overview of the density data of pure [C_4mim]Cl and pure [C_4mim][PF_6] available in the literature at atmospheric pressure (NP: number of data points).

Ionic Liquid	Temperature range (K)	Density range (g · cm^{-3})	NP	Reference
[C_4mim][PF_6]	293.5-414.9	1.370-1.270	10	[23]
	278.2-413.2	1.383-1.274	10	[24]
	293.2-363.2	1.372-1.315	15	[25]
[C_4mim]Cl	308.2-373.2	1.079-1.044	14	[5]
	313.1-364.8	1.073-1.045	11	[32]
	303.1-363.1	1.080-1.047	13	[31]
	348.2-373.2	1.054-1.041	6	[34]
	348.2-373.2	1.056-1.043	6	[35]
	298.2-333.2	1.082-1.045	4	[28]
	298.2-328.2	1.074-1.056	5	[27]
	343.2-363.2	1.058-1.047	5	[33]
	298.2-318.2	1.082-1.071	3	[30]
	298.2-313.2	1.075-1.063	4	[29]

Table S6. Densities (ρ / g·cm^{-3}) for the [C_4mim][PF_6]/[C_4mim]Cl equimolar mixture (estimated).[a]

T / K	[C_4mim][PF_6]/[C_4mim]Cl
298.15	1.240
303.15	1.237
313.15	1.231
323.15	1.225
333.15	1.218
343.15	1.212
353.15	1.204
363.15	1.198

[a]The uncertainties in temperature and density are: $u(T) = \pm$ 0.02 K, and $u(\rho) = \pm 5 \times 10^{-4}$ g·cm^{-3}.

Table S7. Gas–liquid partition coefficients, K_L, of the solutes in $[C_4mim][PF_6]$, $[C_4mim][PF_6]/[C_4mim]Cl$ equimolar mixture, and $[C_4mim]Cl$ [5,6].[a]

Solute	$[C_4mim][PF_6]$[b]						$[C_4mim][PF_6]$/[C_4mim]Cl equimolar mixture[c]						$[C_4mim]Cl$[d,e]					
***T* / K**	**333.15**	**343.15**	**353.15**	**363.15**	**373.15**	**383.15**	**333.15**	**343.15**	**353.15**	**363.15**	**373.15**	**383.15**	**333.15**	**343.15**	**353.15**	**363.15**	**373.15**	**383.15**
Heptane	3.10	2.49	2.02	1.60	1.40	1.26	-	-	-	-	-	-	-	-	-	-	-	-
Octane	6.45	4.80	3.59	2.84	2.36	1.89	2.87	2.19	1.66	1.48	-	-	3.70	2.86	2.24	1.78	1.43	1.16
Nonane	12.96	9.28	6.91	5.08	4.02	3.07	6.48	4.56	3.33	2.65	2.05	1.65	8.12	5.52	3.84	2.72	1.96	1.44
Decane	25.55	17.15	12.32	8.94	9.01	6.59	13.86	9.26	6.58	4.99	3.63	2.74	12.09	8.44	6.02	4.37	3.22	2.42
Cyclohexane	5.68	4.64	3.75	3.12	2.69	2.31	1.97	1.83	1.69	1.60	-	-	4.88	3.97	3.27	2.73	2.30	1.95
Methylcyclohexane	7.75	6.16	4.79	3.96	3.29	2.77	3.00	2.51	2.11	1.92	-	-	5.99	4.76	3.82	3.11	2.56	2.13
Benzene	135.68	97.89	72.68	54.79	42.19	32.91	95.75	70.59	53.54	41.50	32.42	26.05	75.65	54.98	40.68	30.60	23.38	18.11
Toluene	244.94	170.27	121.34	88.64	65.97	50.19	167.07	117.36	84.83	64.48	49.46	37.96	123.41	85.76	60.83	43.98	32.35	24.18
Ethylbenzene	340.11	227.06	155.79	110.12	79.79	58.92	233.28	157.40	110.92	80.98	58.68	43.33	175.26	118.32	81.67	57.54	41.30	30.17
p-xylene	437.29	287.67	198.67	139.80	101.72	74.47	290.86	196.04	133.82	95.92	70.85	52.53	184.36	123.89	85.15	59.75	42.72	31.09
Methyl acetate	98.50	72.23	53.42	40.91	31.52	24.63	55.73	42.36	32.38	25.47	20.21	16.37	33.03	24.67	18.73	14.44	11.28	8.94
Ethyl acetate	124.88	89.05	64.73	48.14	36.43	27.46	65.28	47.83	36.05	27.72	21.84	17.45	33.72	24.78	18.53	14.08	10.85	8.48
Vinyl acetate	121.33	86.77	63.60	47.60	36.34	28.25	72.71	53.68	40.53	31.22	24.41	19.61	48.73	35.25	25.97	19.46	14.81	11.43
THF	106.04	77.89	58.05	44.32	34.45	27.11	60.70	45.75	35.11	27.94	22.08	18.31	35.72	27.03	20.79	16.21	12.82	10.26
1,4-dioxane	590.75	400.44	279.13	199.17	144.38	107.11	381.14	262.56	184.71	134.23	99.73	75.69	214.11	148.61	105.31	76.05	55.89	41.74
Diethyl ether	7.74	6.03	4.82	3.93	3.29	2.81	2.67	2.22	1.92	-	-	-	3.77	3.07	2.53	2.11	1.78	1.51
Acetonitrile	544.69	391.32	290.88	219.01	167.82	130.77	419.35	305.55	226.64	171.36	132.59	104.41	361.27	254.56	182.97	133.92	99.68	75.34
Pyridine	1100.38	709.15	484.55	331.03	234.76	167.19	844.14	557.50	376.74	264.52	187.46	136.10	906.90	588.91	391.88	266.69	185.28	131.19
Thiophene	215.50	153.34	112.26	83.92	63.98	49.78	198.35	142.81	104.31	78.28	60.17	46.84	54.74	74.51	103.20	74.51	54.74	40.87
Acetone	168.65	121.65	91.73	69.87	54.11	41.92	94.87	70.84	53.96	41.69	33.25	26.61	52.36	38.95	29.46	22.63	17.63	13.91
2-butanone	240.02	169.77	124.07	91.80	69.22	52.99	135.50	98.16	72.97	55.54	42.91	33.90	-	-	-	-	-	-
Methanol	82.52	60.67	44.87	34.78	27.53	21.49	879.32	584.87	395.50	275.40	197.59	138.71	1955.12	1289.10	870.25	600.34	422.47	302.80
Ethanol	110.36	80.95	59.45	46.65	37.31	29.11	887.56	582.54	396.32	277.57	201.57	145.14	1655.20	1071.79	711.32	482.86	334.65	236.42
1-propanol	188.71	134.05	95.54	72.28	55.74	42.45	1551.15	984.73	644.35	439.55	306.23	215.63	2806.67	1750.49	1121.34	736.16	494.31	338.89
2-propanol	108.17	78.19	57.67	-	34.23	26.53	645.72	422.55	282.05	195.55	140.33	102.92	1066.14	686.01	452.57	305.49	210.60	148.03
Isobutanol	346.04	236.06	166.45	121.14	89.75	67.14	-	1716.71	1081.52	705.75	478.15	327.83	4868.88	2930.47	1815.24	1154.48	752.27	501.27
1-butanol	183.22	127.22	91.56	67.90	50.50	38.64	1048.17	660.92	429.19	286.83	200.31	142.13	1625.18	1018.63	655.58	432.28	291.48	200.62
2-butanol	235.69	163.99	116.51	85.07	63.33	47.94	-	1188.52	758.10	502.77	344.49	242.99	3228.93	1979.75	1247.94	806.89	534.05	361.17
tert-butanol	92.64	65.78	47.60	35.29	26.86	20.91	381.97	247.50	165.82	114.24	81.52	59.38	626.49	402.73	265.44	179.02	123.31	86.61
Water	232.15	170.92	131.36	100.53	78.26	63.57	5637.67	-	2193.85	1467.39	1009.63	697.82	23903.05	14085.83	8553.00	5338.11	3416.87	2238.64
**Terpenes/terpenoids**	**353.15**	**363.15**	**373.15**	**383.15**	**393.15**	**403.15**	**353.15**	**363.15**	**373.15**	**383.15**	**393.15**	**403.15**	**353.15**	**363.15**	**373.15**	**383.15**	**393.15**	**403.15**
α-pinene	26.97	20.23	15.40	12.04	9.59	7.75	17.02	15.50	11.71	8.71	6.70	5.89	4.44	3.41	2.66	2.1	1.68	1.35
β-pinene	59.84	44.56	32.08	24.53	18.25	14.32	40.41	32.61	23.83	19.08	14.94	12.34	9.81	7.25	5.44	4.15	3.2	2.51
Limonene	102.03	71.16	51.41	37.52	28.02	21.26	74.04	54.29	37.40	29.10	23.24	17.18	-	-	-	-	-	-
Myrcene	91.27	61.76	43.34	31.30	23.16	17.60	63.05	42.09	32.36	22.97	17.77	13.77	-	-	-	-	-	-
γ-terpinene	314.04	210.56	145.13	103.10	75.05	55.73	204.75	143.56	103.18	70.05	52.95	39.21	-	-	-	-	-	-
p-cymene	140.62	96.38	67.66	48.63	35.69	26.67	103.47	71.68	51.99	37.87	27.49	20.68	-	-	-	-	-	-

Eucalyptol	186.30	132.14	90.56	65.23	47.84	35.20	109.60	79.77	59.87	40.36	31.02	23.84	19.17	13.76	10.06	7.47	5.63	4.31
**Low volatile terpenoids**	**383.15**	**393.15**	**403.15**	**413.15**	**423.15**	**433.15**	**383.15**	**393.15**	**403.15**	**413.15**	**423.15**	**433.15**	**383.15**	**393.15**	**403.15**	**413.15**	**423.15**	**433.15**
α-pinene oxide	270.43	190.74	139.98	105.07	79.50	62.12	165.62	120.27	88.66	67.90	52.62	39.35	36.41	25.77	18.56	13.59	10.1	7.61
Fenchone	410.12	291.03	210.22	155.91	118.06	90.09	239.75	171.06	120.52	94.54	71.18	53.25	46.22	32.92	23.85	17.56	13.13	9.94
Menthone	507.24	352.24	249.38	180.61	134.16	101.87	295.31	205.98	147.54	109.59	83.26	64.56	63.63	43.7	30.59	21.79	15.79	11.61
Carvone	1854.64	1230.15	836.01	579.42	413.90	299.54	1270.71	860.46	577.97	409.92	287.44	204.74	372.3	242.83	161.87	110.09	76.29	53.79
Isopulegol	541.77	368.76	257.82	184.10	136.45	101.19	1422.81	889.88	596.52	403.67	270.42	188.94	977.43	597.61	374.64	240.37	157.57	105.37
Citronellol	890.40	565.37	-	263.39	186.51	133.78	3959.04	2349.61	1425.46	918.23	607.59	409.36	3192.98	1828.23	1076.92	651.24	403.53	255.76
Geraniol	1379.30	900.40	590.07	399.07	281.99	199.72	-	3845.17	2367.00	1465.05	935.95	611.33	5610.21	3158.11	1830.5	1090.09	665.66	416.07
Linalool	348.89	238.71	163.47	115.57	83.09	59.89	1136.43	706.95	445.72	296.35	202.39	131.23	845.75	503.77	308.08	193.06	123.75	81.01
Citronellal	439.90	300.53	209.29	148.95	109.58	82.7	277.42	195.44	139.69	110.21	79.98	-	-	-	-	-	-	-
Camphor	768.10	537.13	380.46	275.89	204.77	153.55	-	319.26	231.06	173.15	125.68	94.98	104.4	71.6	50.05	35.62	25.77	18.93
Borneol	712.01	476.42	331.39	240.37	174.09	128.03	3041.14	1864.09	1218.97	815.52	544.07	-	2760.58	1635.77	995.42	620.85	396.19	258.24
Menthol	514.57	345.54	234.07	166.99	121.17	89.13	1761.51	1079.08	688.30	463.36	305.72	209.09	1412.22	834.49	506.49	315.12	200.61	130.47
**Phenolic Terpenoids**	**413.15**	**423.15**	**433.15**	**443.15**	**453.15**													
Eugenol	2793.29	1839.16	1276.32	876.73	624.55													
Carvacrol	2007.14	1327.99	896.86	624.45	441.41													
Thymol	1548.37	1034.42	700.96	488.02	350.61													

[a]The estimated uncertainties in the pressure and temperature are $u(T)$ = 0.1 K, and $u(p)$ = 0.05.
[b]Colunm Packing: 50% of IL, $n_3 = 9.48$ mmol.
[c]Colunm Packing: 50% of IL, $n_3 = 10.75$ mmol.
[d]Colunm Packing: 51.1% of IL, $n_3 = 17.02$ mmol.
[e]Extrapolated using the data reported by the authors [5,6].

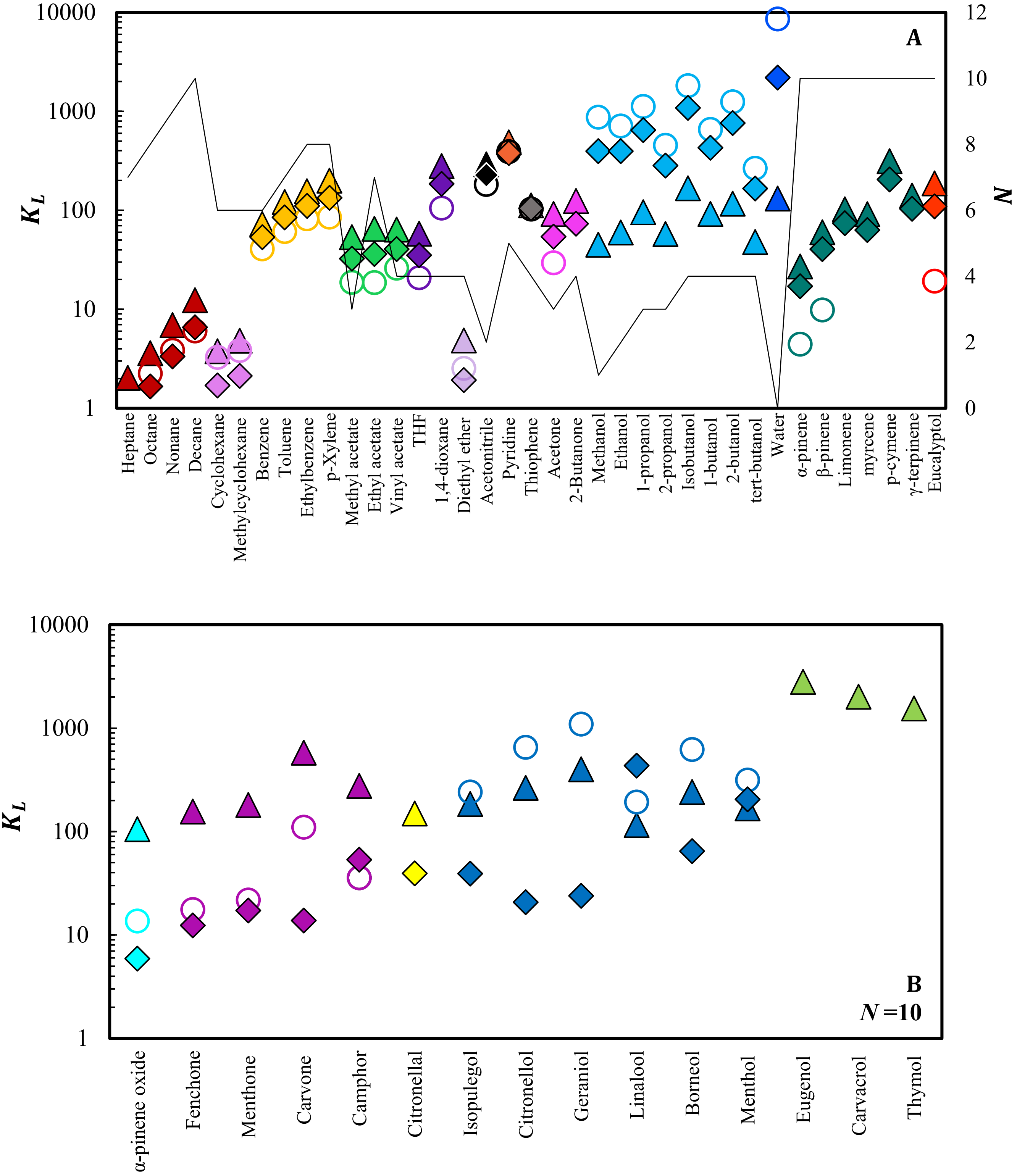

Figure S3. Gas–liquid partition coefficients (K_L) of **A**, common organic solutes, water, hydrocarbon terpenes and eucalyptol at 353.15 K and **B**, less-volatile terpenoids at 413.2 K. Symbols code: (○), $[C_4mim]Cl$ [5,6]; (◆), $[C_4mim][PF_6]/[C_4mim]Cl$ equimolar mixture; and (▲), $[C_4mim][PF_6]$. Different colors symbols represent different chemical families while the solid line indicates the number of carbons (N) present in each solute. On panel **B**, all solutes have 10 carbons atoms.

Limiting partial molar excess properties

Table S8. Thermodynamic functions at infinite dilution: partial molar excess Gibbs free energies ($\bar{G}_m^{E,\infty}$/kJ·mol^{-1}), partial molar excess enthalpies ($\bar{H}_m^{E,\infty}$/kJ·mol^{-1}), and partial molar excess entropies ($T_{ref}\bar{S}_m^{E,\infty}$/kJ·mol^{-1}) of several solutes in [C_4mim][PF_6], equimolar [C_4mim][PF_6]/[C_4mim]Cl mixture, and [C_4mim]Cl [5,6].

Solutes	[C_4mim][PF_6]			[C_4mim][PF_6]/[C_4mim]Cl equimolar mixture			[C_4mim]Cl[a,b]		
	$\bar{G}_m^E$	$\bar{H}_m^E$	$T_{ref}\bar{S}_m^E$	$\bar{G}_m^E$	$\bar{H}_m^E$	$T_{ref}\bar{S}_m^E$	$\bar{G}_m^E$	$\bar{H}_m^E$	$T_{ref}\bar{S}_m^E$
	T_{ref} = 353.2 K								
Heptane	14.14	11.33	-2.82	-	-	-	-	-	-
Octane	15.03	9.72	-5.31	17.64	13.00	-4.64	17.16	9.94	-7.21
Nonane	15.64	9.64	-6.00	18.14	11.26	-6.88	18.21	9.34	-8.88
Decane	16.51	10.14	-6.37	18.71	10.48	-8.23	19.35	9.07	-10.28
Cyclohexane	10.70	8.81	-1.89	13.39	21.21	7.81	11.85	7.67	-4.19
Methylcyclohexane	11.71	8.35	-3.36	14.47	15.59	1.12	13.12	7.42	-5.70
Benzene	1.93	-1.34	-3.27	3.18	0.98	-2.20	4.38	-2.62	-7.00
Toluene	3.20	-0.87	-4.07	4.61	1.49	-3.12	5.97	-2.74	-8.71
Ethylbenzene	4.90	-0.49	-5.39	6.24	1.02	-5.23	7.53	-1.72	-9.25
p-xylene	4.40	-0.47	-4.87	5.88	0.78	-5.10	7.63	-1.86	-9.49
Methyl acetate	0.77	-2.61	-3.38	2.59	0.65	-1.95	4.58	-2.17	-6.75
Ethyl acetate	2.03	-1.64	-3.67	4.11	1.83	-2.27	6.45	-0.58	-7.03
Vinyl acetate	1.67	-1.64	-3.30	3.34	1.35	-1.99	5.04	-2.54	-7.58
THF	1.32	-1.75	-3.08	0.88	0.77	-0.11	5.08	-0.24	-5.33
1,4-dioxane	0.00	-3.68	-3.68	1.61	-1.79	-3.40	3.61	-2.92	-6.52
Diethyl ether	6.08	1.10	-4.98	9.24	5.09	-4.15	8.71	1.99	-6.72
Acetonitrile	-1.87	-1.37	0.50	-0.79	-0.80	-0.01	0.24	-5.10	-5.35
Pyridine	-0.35	-4.94	-4.59	0.74	-3.87	-4.61	1.01	-7.27	-8.27
Thiophene	1.01	-1.64	-2.65	-0.77	-0.22	0.54	2.00	-6.35	-8.35
Acetone	-0.85	-2.93	-2.08	1.07	-0.65	-1.72	3.23	-2.59	-5.83
2-butanone	0.33	-2.66	-2.98	2.24	-0.13	-2.37	-	-	-
Methanol	1.67	5.05	3.38	-4.17	-5.38	-1.21	-6.53	-9.42	-2.88
Ethanol	2.32	8.98	6.66	-2.85	-1.30	1.56	-4.50	-8.17	-3.67
1-propanol	0.00	8.84	8.84	-2.06	-1.31	0.76	-3.74	-9.66	-5.93
2-propanol	2.86	8.61	5.75	-1.42	-0.63	0.79	-2.90	-9.85	-6.95
Isobutanol	3.81	9.45	5.65	-1.31	-0.22	1.09	-2.95	-9.85	-6.90

1-butanol	3.93	10.25	6.32	-1.17	-0.73	0.44	-2.78	-9.35	-6.57
2-butanol	3.52	8.00	4.48	-0.59	-1.18	-0.59	-2.02	-10.28	-8.26
tert-butanol	3.45	5.86	2.40	0.24	-1.95	-2.20	-1.39	-11.84	-10.45
Water	2.33	11.67	9.34	-5.40	-5.20	0.20	-9.39	-13.93	-4.53
**Terpenes and Terpenoids**					**T_{ref} = 383.2 K**				
α-pinene	12.26	5.97	-6.29	13.75	8.55	-5.20	18.27	7.96	-10.31
β-pinene	10.92	4.15	-6.77	12.08	10.08	-2.00	16.98	4.66	-12.32
Limonene	10.77	4.57	-6.20	12.03	8.06	-3.97	-	-	-
Myrcene	10.82	1.24	-9.59	12.22	4.54	-7.68	-	-	-
γ-terpinene	10.10	1.09	-9.02	11.32	2.29	-9.02	-	-	-
p-cymene	8.08	1.00	-7.08	9.73	2.52	-7.22	-	-	-
Eucalyptol	8.86	3.37	-5.49	10.88	6.71	-4.18	16.20	3.41	-12.79
α-pinene oxide	5.84	3.16	-9.71	13.75	-1.64	-9.44	14.08	-5.09	-19.17
Fenchone	5.50	-3.87	-6.15	7.79	-0.03	-7.63	13.35	3.20	-10.15
Menthone	6.11	-0.66	-7.21	7.60	1.24	-6.98	14.41	0.64	-13.77
Carvone	3.91	-1.10	-7.24	8.22	-3.45	-8.96	11.43	-1.55	-12.98
Isopulegol	6.21	-3.33	-3.92	5.51	-7.09	-7.09	6.31	-7.76	-14.07
Citronellol	7.67	2.29	0.48	0.00	-2.47	-5.78	6.09	-8.80	-14.89
Geraniol	6.92	8.15	0.31	3.31	-5.24	-5.24	4.86	-12.26	-17.13
Linalool	6.71	7.23	-5.96	0.00	-9.73	-13.08	12.58	-42.92	-55.50
Citronellal	6.82	0.75	-4.15	3.35	8.04	-0.64	-	-	-
Camphor	4.70	2.67	-7.57	8.68	-1.26	-1.26	12.54	-0.16	-12.70
Borneol	7.63	-2.86	5.08	0.00	2.53	-0.87	4.63	13.18	8.55
Menthol	7.79	12.71	-3.04	3.40	-4.94	-9.21	6.94	-8.55	-15.49
**Phenolic Terpenoids**					**T_{ref} = 413.2 K**				
Eugenol	4.01	-9.59	-13.60	-	-	-	-	-	-
Carvacrol	2.02	-9.55	-11.57	-	-	-	-	-	-
Thymol	2.10	-5.81	-7.92	-	-	-	-	-	-

[a]The partial molar thermodynamic functions of water and organic solutes in [C_4mim]Cl were calculated from the γ_{13}^{∞} data obtained in the temperature range of 358.15-388.15 K, reported by Martins et al. [5].

[b]The partial molar thermodynamic functions of terpenes and terpenoids in [C_4mim]Cl were calculated from the γ_{13}^{∞} data obtained in the temperature range of 398.15-448.15 K, reported by Martins et al. [6].

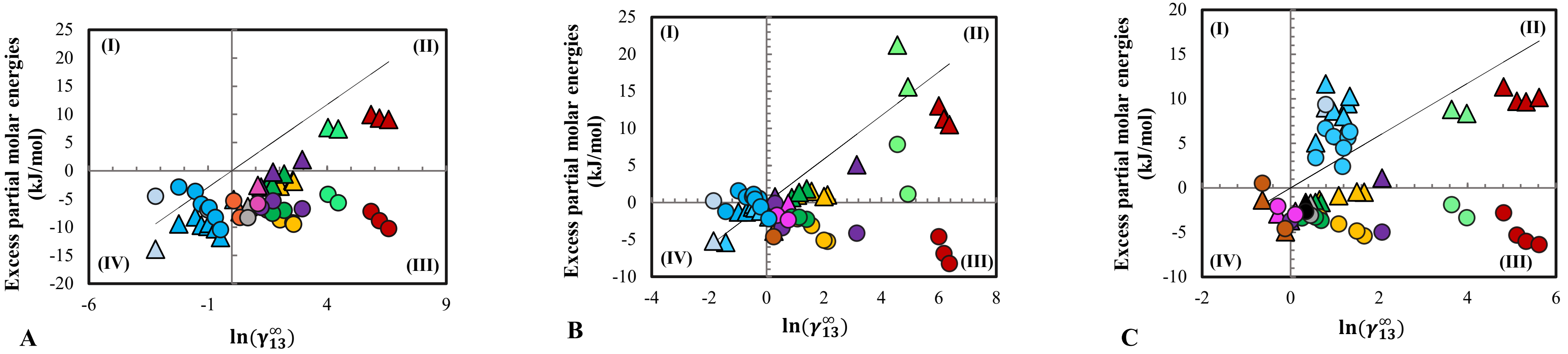

Figure S4. Partial molar excess properties at 353.15 K of the studied organic solutes as a function of ln (γ_{13}^{∞}) for **A**, [C4mim]Cl (interpolated values from [5]); **B**, [C4mim]Cl/[C4mim][PF6] equimolar mixture; and **C**, [C4mim][PF6]. The solid line represents $\bar{G}_m^{E,\infty}$, the triangles correspond to $\bar{H}_m^{E,\infty}$, and the circles are $T_{ref}\bar{S}_m^{E,\infty}$. Color code: ●, alkanes; ●, cycloalkanes; ●, aromatic hydrocarbons; ●, ethers; ●, esters; ●, ketones; ●, alcohols; ●, water; ●, acetonitrile and pyridine; and ●, thiophene.

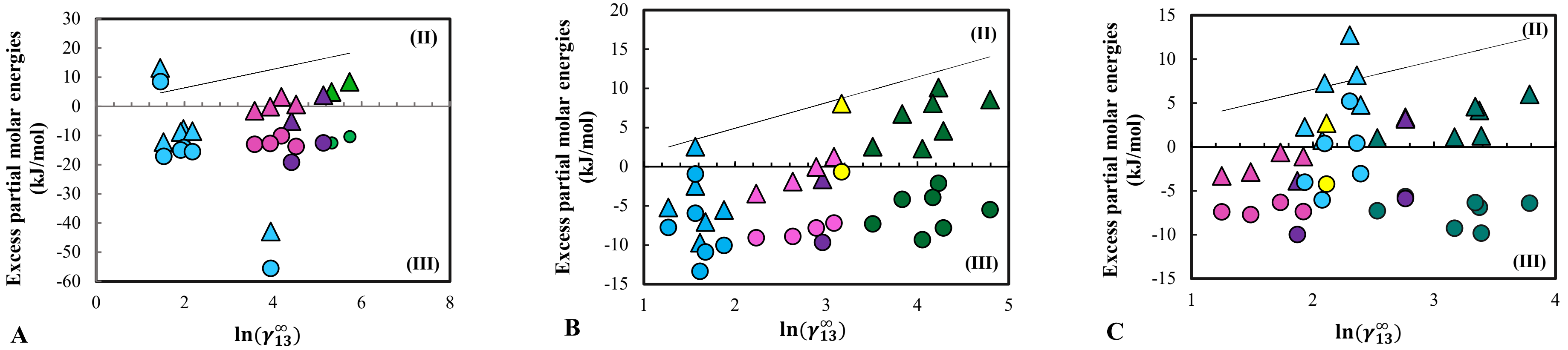

Figure S5. Partial molar excess properties at 393.15 K of the studied terpenes and terpenoids as a function of ln (γ_{13}^{∞}) for **A**, [C4mim]Cl (interpolated values from [6]); **B**, [C4mim]Cl/[C4mim][PF6] equimolar mixture; and **C**, [C4mim][PF6]. The solid line represents $\bar{G}_m^{E,\infty}$, the triangles correspond to $\bar{H}_m^{E,\infty}$, and the circles are $T_{ref}\bar{S}_m^{E,\infty}$. Color code: ●, alcohol terpenoids; ●, ketone terpenoids; ●, ether terpenoids; ●, citronellal; ●, hydrocarbon terpenes.

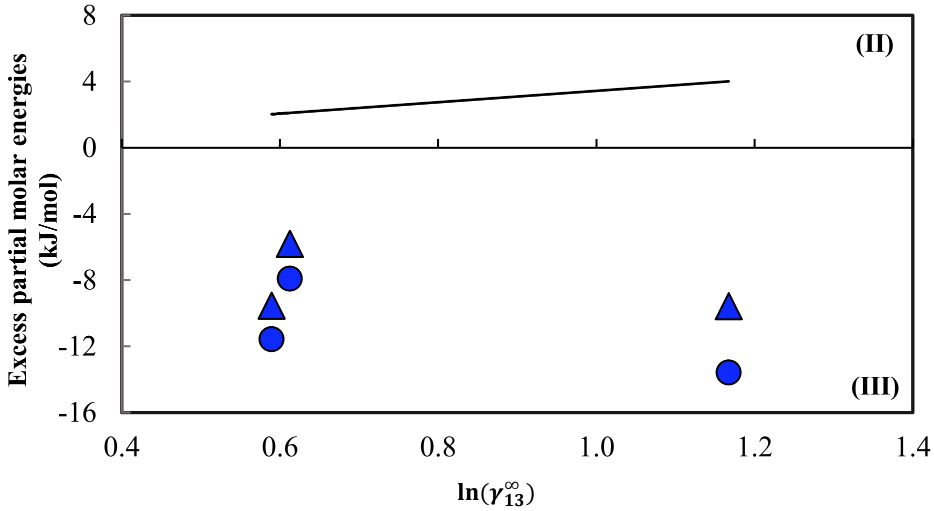

Figure S6. Partial molar excess properties as a function of $\ln(\gamma_{13}^{\infty})$ for phenolic terpenoids in $[C_4mim][PF_6]$ at 413.2 K. The solid line represents $\bar{G}_m^{E,\infty}$, the triangles correspond to $\bar{H}_m^{E,\infty}$, and the circles are the $T_{ref}\bar{S}_m^{E,\infty}$.

Fractionation factors

Table S9. Selectivities (S_{ij}^{∞}), capacities (k_j^{∞}), and solvent performance indexes (Q_{ij}^{∞}) at infinite dilution for different terpenes mixtures in ILs at 403.2 K.[a]

Ionic Liquid	$S_{ij}^{\infty}/k_j^{\infty}/Q_{ij}^{\infty}$							Source
	α-pinene/β-pinene	β-pinene/limonene	*p*-cymene/limonene	limonene/linalool	menthol/menthone	borneol/camphor	limonene/carvone	
$[C_4mim][PF_6]$	1.49/0.03/0.05	1.04/0.04/0.04	1.25/0.08/0.10	3.46/0.13/0.43	1.55/0.14/0.22	2.00/0.22/ 0.44	7.71/0.28/2.16	This work
$[C_4mim][PF_6]$/ $[C_4mim]Cl$	1.69/0.03/0.04	1.02/0.03/0.03	1.93/0.05/0.10	11.73/0.30/3.54	3.21/0.24/0.78	3.02/0.36/ 1.08	6.63/0.17/1.13	This work
$[C_4mim]Cl$	1.41/0.01/0.01	-	-	-	9.05/0.89/8.05	14.77/4.24/62.62	-	[6],[b]
$[P_{6,6,6,14}]Cl$	1.14/0.99/1.13	1.12/0.99/1.11	1.04/0.88/0.92	10.20/9.01/91.88	10.16/11.72/119.14	7.48/14.15/105.90	1.23/1.09/1.34	[7]
$[P_{6,6,6,14}][(C_8H_{17})_2PO_2]$	1.07/1.59/1.69	1.15/1.59/1.82	1.03/1.38/1.42	5.93/8.20/ 48.64	-	-	1.24/1.38/1.71	
$[C_4mim][OAc]$	1.33/0.06/0.08	1.35/0.06/0.08	1.62/0.07/0.11	-	-	-	5.38/0.23/1.21	
$[C_8mim]Cl$	1.37/0.08/0.11	1.07/0.09/0.09	1.34/0.12/0.15	15.40/1.34/ 20.61	11.62/1.76/20.43	16.86/3.21/54.11	3.46/0.30/1.04	[16]
$[C_4mim]Cl/[C_{12}mim]Cl$	1.38/0.11/0.15	1.04/0.11/0.11	1.22/0.13/0.16	14.06/1.54/21.62	11.79/1.97/23.21	18.11/3.28/59.32	3.07/0.34/1.03	
$[C_{12}mim]Cl$	1.24/0.24/0.29	1.01/0.24/0.29	1.06/0.25/0.27	9.65/2.27/ 21.88	8.48/2.39/20.25	12.54/4.04/ 50.64	1.81/0.43/0.77	

[a] j = solute with the lowest activity coefficient in each separation pair.
[b] Interpolated using the data reported by the authors.

Table S10. Selectivities (S_{ij}^{∞}), capacities (k_j^{∞}), and solvent performance indexes (Q_{ij}^{∞}) at infinite dilution for selected mixtures involving phenolic terpenoids in [C_4mim][PF_6] and [C_4mim][CF_3SO_3] at 413.2 K.

Mixture	Ionic liquid	S_{ij}^{∞}	k_j^{∞}	Q_{ij}^{∞}	Source
linalool/thymol	[C_4mim][PF_6]	4.75	0.54	2.57	This work
	[C_4mim][CF_3SO_3]	113.53[a]	1.57[a]	178.22[a]	[6]
linalool/eugenol	[C_4mim][PF_6]	2.73	0.31	0.85	This work
	[C_4mim][CF_3SO_3]	55.58[a]	0.77[a]	42.7[a]	[6]
eucalyptol/eugenol	[C_4mim][PF_6]	4.73[b]	0.31	1.47[b]	This work
	[C_4mim][CF_3SO_3]	9.47[a]	0.77[a]	7.28[a]	[6]
α-pinene/carvacrol	[C_4mim][PF_6]	22.82[b]	0.55	12.66[b]	This work
	[C_4mim][CF_3SO_3]	35.25[a]	1.58[a]	55.83[a]	[6]
α-pinene/thymol	[C_4mim][PF_6]	22.30[b]	0.54	12.09[b]	This work
	[C_4mim][CF_3SO_3]	34.94	1.57	54.85	[6]
β-pinene/carvacrol	[C_4mim][PF_6]	15.22[b]	0.55	8.44[b]	This work
	[C_4mim][CF_3SO_3]	27.38[a]	1.58[a]	43.36[a]	[6]
β-pinene/thymol	[C_4mim][PF_6]	14.88[b]	0.54	8.07[b]	This work
	[C_4mim][CF_3SO_3]	27.14[a]	1.57[a]	42.60[a]	[6]
γ-terpinene/carvacrol	[C_4mim][PF_6]	6.90[b]	0.55	3.80[b]	This work
γ-terpinene/thymol		6.74[b]	0.54	3.65[b]	
myrcene/carvacrol		16.19[b]	0.55	8.98[b]	
myrcene/thymol		15.83[b]	0.54	8.58[b]	
limonene/carvacrol		14.74[b]	0.55	8.17[b]	
limonene/thymol		14.40[b]	0.54	7.81[b]	
p-cymene/carvacrol		13.01[b]	0.55	7.21[b]	
p-cymene/thymol		12.71[b]	0.54	6.89[b]	

[a]Interpolated values using the data reported by the authors.
[b]Extrapolated values using the experimental data measured in this work.

Table S11. Selectivities (S_{ij}^{∞}), capacities (k_j^{∞}), and solvent performance indexes (k_j^{∞}) at infinite dilution for selected mixtures of organic compounds relevant in fuel separation problems, in methylimidazolium-based ionic liquids, at 333.2 K.

Ionic Liquid	S_{ij}^{∞} / k_j^{∞} / Q_{ij}^{∞}				Source
	octane/benzene	cyclohexane/benzene	octane/thiophene	octane/pyridine	
[C_4mim][PF_6]	103.77/0.53/55.02	24.11/0.53/12.78	143.39/0.73/105.07	241.90/1.24/299.02	This work
	42.56/0.47/19.98	9.38/0.47/4.40	56.66/0.63/35.41	122.51/1.35/165.56	[18]
[C_4mim][PF_6]/[C_4mim]Cl	164.36/0.33/54.55	49.00/0.33/16.17	296.25/0.60/177.22	416.53/0.84/350.34	This work
[C_4mim]Cl	100.36/0.24/23.83	15.72/0.24/3.77	243.46/0.58/140.22	347.99/0.82/286.43	[5],[a]
[C_4mim][SCN]	93.56/0.58/54.72	16.49/0.58/9.64	156.86/0.98/153.79	-	[36],[b]
[C_4mim][CF_3SO_3]	38.36/0.63/24.13	10.18/0.63/6.41	52.58/0.86/45.33	-	[37],[b]
[C_4mim][DCA]	63.31/0.50/31.82	12.38/0.50/6.22	100.40/0.80/80.00	137.70/0.92/126.00	[38],[b]
[C_4mim][BETI]	18.51/0.98/18.26	6.45/0.98/6.36	19.25/1.02/19.74	46.00/2.45/112.76	[39],[b]
[C_4mim][OAc]	33.28/0.39/12.95	7.88/0.39/3.07	69.54/0.81/56.54	67.88/0.79/53.88	[7]
[C_4mim][CH_3SO_3]	55.46/0.32/18.01	5.99/0.32/1.94	99.32/0.58/57.75	15.63/0.09/1.43	[5],[a]
[C_4mim][$(CH_3)_2PO_4$]	20.55/0.43/8.82	5.27/0.43/2.26	38.62/0.81/31.15	40.59/0.85/34.40	[5],[a]
[C_4mim][TOS]	14.79/0.51/7.55	8.32/0.51/4.24	57.57/0.90/51.86	-	[40],[b]
[C_4mim][TCM]	45.35/0.77/34.88	10.19/0.77/7.84	62.84/1.07/67.00	96.79/1.64/158.95	[41],[b]
[C_4mim][DBP]	6.83/1.07/7.30	3.05/1.07/3.30	-	-	[42]
[C_8mim]Cl	27.91/0.48/13.45	19.80/0.48/9.54	53.62/0.93/49.65	59.51/1.03/61.17	
[C_4mim]Cl/[C_{12}mim]Cl	13.06/0.50/6.56	10.34/0.50/5.19	23.96/0.92/22.06	23.17/0.89/20.63	[16]
[C_{12}mim]Cl	4.91/0.82/4.04	3.27/0.82/2.70	7.38/1.24/9.13	5.77/0.97/5.59	

[a]Extrapolated using the data reported by the authors.
[b]Interpolated using the data reported by the authors.

Table S12. Selectivities (S_{ij}^{∞}) at infinite dilution for selected mixtures of organic compounds in eutectic mixtures [43] and in $[C_4mim]Cl/[C_4mim][PF_6]$ equimolar mixture at 298.2 K.

	decane/butanol	decane/benzene	ethyl acetate/butanol	cyclohexane/benzene	Reference
[Ch]Cl + Glycerol (1:1)	90	31	3	8.4	[43]
[Ch]Cl + Glycerol (1:2)	62	27	2.1	6.4	[43]
$[C_4mim][PF_6]/[C_4mim]Cl$	1653	287	8	72	This work

References

1. Paulechka, Y.U.; Kohut, S. V.; Blokhin, A. V.; Kabo, G.J. Thermodynamic Properties of 1-Butyl-3-Methylimidazolium Trifluoromethanesulfonate Ionic Liquid in the Condensed State. *Thermochim. Acta* **2010**, *511*, 119–123, doi:10.1016/j.tca.2010.08.002.
2. Domańska, U.; Bogel-Łukasik, E.; Bogel-Łukasik, R. 1-Octanol/Water Partition Coefficients of 1-Alkyl-3-Methylimidazolium Chloride. *Chem. – A Eur. J.* **2003**, *9*, 3033–3041, doi:10.1002/chem.200204516.
3. Yaws, C.L. *Thermophysical Properties of Chemicals and Hydrocarbons*; 2nd ed.; Elsevier Inc., 2014;
4. Pence, H.E.; Williams, A. ChemSpider: An Online Chemical Information Resource. *J. Chem. Educ.* **2010**, *87*, 1123–1124, doi:10.1021/ed100697w.
5. Martins, M.A.R.; Coutinho, J.A.P.; Pinho, S.P.; Domańska, U. Measurements of Activity Coefficients at Infinite Dilution of Organic Solutes and Water on Polar Imidazolium-Based Ionic Liquids. *J. Chem. Thermodyn.* **2015**, *91*, 194–203, doi:10.1016/j.jct.2015.07.042.
6. Martins, M.A.R.; Domańska, U.; Schröder, B.; Coutinho, J.A.P.; Pinho, S.P. Selection of Ionic Liquids to Be Used as Separation Agents for Terpenes and Terpenoids. *ACS Sustain. Chem. Eng.* **2016**, *4*, 548–556, doi:10.1021/acssuschemeng.5b01357.
7. Vilas-Boas, S.M.; Teixeira, G.; Rosini, S.; Martins, M.A.R.; Gaschi, P.S.; Coutinho, J.A.P.; Ferreira, O.; Pinho, S.P. Ionic Liquids as Entrainers for Terpenes Fractionation and Other Relevant Separation Problems. *J. Mol. Liq.* **2021**, *323*, 114647, doi:10.1016/j.molliq.2020.114647.
8. Everett, D.H. Effect of Gas Imperfection on G.L.C. Measurements : A Refined Method for Determining Activity Coefficients and Second Virial Coefficients. *Trans. Faraday Soc.* **1965**, *61*, 1637–1645, doi:10.1039/TF9656101637.
9. Cruickshank, A.J.B.; Gainey, B.W.; Hicks C. P.; Letcher, T.M.; Moody, R.W.; Young, C.L. Gas-Liquid Chromatographic Determination of Cross-Term. *Trans. Faraday Soc.* **1967**, *65*, 1014–1031, doi:10.1039/tf9696501014.
10. Tsonopoulos, C. An Empirical Correlation of Second Virial Coefficients. *AIChE J.* **1974**, *20*, 263–272, doi:10.1002/aic.690200209.
11. Poling, B.E.; Prausnitz, J.M. *Properties of Gases and Liquids*; McGraw-Hill Publishing, 2001.
12. Vilas-Boas, S.M.; Martins, M.A.R.; Tentor, F.R.; Teixeira, G.; Sgorlon, J.G.; Coutinho, J.A.P.; Ferreira, O.; Pinho, S.P. Imidazolium Chloride Ionic Liquid Mixtures as Separating Agents: Fuel Processing and Azeotrope Breaking. *Energy and Fuels* **2022**, *36*, 8552–8561, doi:10.1021/acs.energyfuels.2c01724.
13. Martins, M.A.R.; Vilas-Boas, S.M.; Cordova, I.W.; Carvalho, P.J.; Domańska, U.; Ferreira, O.; Coutinho, J.A.P.; Pinho, S.P. Infinite Dilution Activity Coefficients in the Smectic and Isotropic Phases of Tetrafluoroborate-Based Ionic Liquids. *J. Chem. Eng. Data* **2021**, *66*, 2587–2596, doi:10.1021/acs.jced.1c00182.
14. Domańska, U.; Wlazło, M.; Karpińska, M. [DCA]-Based Ionic Liquids for the Extraction of Sulfur and Nitrogen Compounds from Fuels: Activity Coefficients at Infinite Dilution. *Fluid Phase*

Equilib. **2020**, *507*, 1–11, doi:10.1016/j.fluid.2019.112424.

15. Castells, R.C. Determination of Gas-Liquid Partition Coefficients by Gas Chromatography. *J. Chromatogr. A* **2004**, *1037*, 223–231, doi:10.1016/j.chroma.2003.12.019.
16. Vilas Boas, S.M.; Zambom, A.; Martins, M.A.R.; Coutinho, J.A.P.; Ferreira, O.; Pinho, S.P. Evaluation of Ionic Liquids for the Sustainable Fractionation of Essential Oils. *Ind. Eng. Chem. Res.* **2022**, Submitted.
17. Boussaha, M.; Khimeche, K.; Dahmani, A. Activity Coefficients at Infinite Dilution for Hydrocarbons in Fatty Alcohols Determined by Gas−Liquid Chromatography. *J. Chem. Eng. Data* **2011**, *56*, 850–858, doi:10.1021/je1005517.
18. Mutelet, F.; Butet, V.; Jaubert, J.-N. Application of Inverse Gas Chromatography and Regular Solution Theory for Characterization of Ionic Liquids. *Ind. Eng. Chem. Res.* **2005**, *44*, 4120–4127, doi:10.1021/ie048806l.
19. Zhu, J.; Yu, Y.; Chen, J.; Fei, W. Measurement of Activity Coefficients at Infinite Dilution for Hydrocarbons in Imidazolium-Based Ionic Liquids and QSPR Model. *Front. Chem. Eng. China* **2007**, *1*, 190–194, doi:10.1007/s11705-007-0035-3.
20. Dobryakov, Y.G.; Tuma, D.; Maurer, G. Activity Coefficients at Infinite Dilution of Alkanols in the Ionic Liquids 1-Butyl-3-Methylimidazolium Hexafluorophosphate, 1-Butyl-3-Methylimidazolium Methyl Sulfate, and 1-Hexyl-3-Methylimidazolium Bis(Trifluoromethylsulfonyl) Amide Using the Dilutor T. *J. Chem. Eng. Data* **2008**, *53*, 2154–2162, doi:10.1021/je800355e.
21. Spange, S.; Lungwitz, R.; Schade, A. Correlation of Molecular Structure and Polarity of Ionic Liquids. *J. Mol. Liq.* **2014**, *192*, 137–143, doi:10.1016/j.molliq.2013.06.016.
22. Ab Rani, M. A.; Brant, A.; Crowhurst, L.; Dolan, A.; Lui, M.; Hassan, N.H.J.; Hallett, P.;Hunt, P. A.; Niedermeyer, H.; Perez-Arlandis, J. M.; Schrems, M.;Welton, T.. W.R. Understanding the Polarity of Ionic Liquids. *Phys. Chem. Chem. Phys.* **2011**, *13*, 16831–16840, doi:10.1039/c1cp21262a.
23. Jacquemin, J.; Husson, P.; Mayer, V.; Cibulka, I. High-Pressure Volumetric Properties of Imidazolium-Based Ionic Liquids : Effect of the Anion. *J. Chem. Eng. Data* **2007**, *52*, 2204–2211, doi:10.1021/je700224j.
24. Safarov, J.; Suleymanli, K.; Aliyev, A.; Yeadon, D.J.; Jacquemin, J.; Bashirov, M.; Hassel, E. (P,ρ,T) Data of 1-Butyl-3-Methylimidazolium Hexafluorophosphate. *J. Chem. Thermodyn.* **2020**, *141*, 1–14, doi:10.1016/j.jct.2019.105954.
25. Rocha, M.A.A.; Ribeiro, F.M.S.; Ferreira, A.I.M.C.L.; Coutinho, J.A.P.; Santos, L.M.N.B.F. Thermophysical Properties of [CN - 1C1im][PF 6] Ionic Liquids. *J. Mol. Liq.* **2013**, *188*, 196–202, doi:10.1016/j.molliq.2013.09.031.
26. Rebelo, L.P.N.; Najdanovic-Visak, V.; de Azevedo, R.G.; Esperança, J.M.S.S.; da Ponte, M.N.; Guedes, H.J.R.; Visak, Z.P.; de Sousa, H.C.; Szydlowski, J.; Lopes, J.N.C.; et al. Phase Behavior and Thermodynamic Properties of Ionic Liquids, Ionic Liquid Mixtures, and Ionic Liquid Solutions. In *ACS Symposium Series*; Rogers, R.D., Seddon, K.R., Eds.; ACS Symposium Series; American Chemical Society: Washington, DC, 2005; Vol. 901, pp. 270–291 ISBN

9780841238930.
27. Govinda, V.; Attri, P.; Venkatesu, P.; Venkateswarlu, P. Thermophysical Properties of Dimethylsulfoxide with Ionic Liquids at Various Temperatures. *Fluid Phase Equilib.* **2011**, *304*, 35–43, doi:http://dx.doi.org/10.1016/j.fluid.2011.02.010.
28. He, R.-H.; Long, B.-W.; Lu, Y.-Z.; Meng, H.; Li, C.-X. Solubility of Hydrogen Chloride in Three 1-Alkyl-3-Methylimidazolium Chloride Ionic Liquids in the Pressure Range (0 to 100) KPa and Temperature Range (298.15 to 363.15) K. *J. Chem. Eng. Data* **2012**, *57*, 2936–2941, doi:10.1021/je3003783.
29. Kavitha, T.; Vasantha, T.; Venkatesu, P.; Rama Devi, R.S.; Hofman, T. Thermophysical Properties for the Mixed Solvents of N-Methyl-2-Pyrrolidone with Some of the Imidazolium-Based Ionic Liquids. *J. Mol. Liq.* **2014**, *198*, 11–20, doi:http://dx.doi.org/10.1016/j.molliq.2014.07.002.
30. Kumar, B.; Singh, T.; Rao, K.S.; Pal, A.; Kumar, A. Thermodynamic and Spectroscopic Studies on Binary Mixtures of Imidazolium Ionic Liquids in Ethylene Glycol. *J. Chem. Thermodyn.* **2012**, *44*, 121–127, doi:10.1016/j.jct.2011.08.008.
31. Dowell, N. Mac; Llovell, F.; Sun, N.; Hallet, J.P.; George, A.; Hunt, P.A.; Welton, T.; Simmons, B.A.; Vega, L.F. New Experimental Density Data and Soft-SAFT Models of Alkylimidazolium ([CnC1im]+) Chloride (Cl−), Methylsulfate ([MeSO4]−), and Dimethylphosphate ([Me2PO4]−) Based Ionic Liquids. *J. Phys. Chem. B* **2014**, *118*, 6206–6221, doi:10.1021/jp501619y.
32. Součková, M.; Klomfar, J.; Pátek, J. Group Contribution and Parachor Analysis of Experimental Data on Density and Surface Tension for Members of the Homologous Series of 1-C -3-Methylimidazolium Chlorides. *Fluid Phase Equilib.* **2017**, *454*, 43–56, doi:10.1016/j.fluid.2017.08.022.
33. Vieira, N.S.M.; Vázquez-Fernández, I.; Araújo, J.M.M.; Plechkova, N. V.; Seddon, K.R.; Rebelo, L.P.N.; Pereiro, A.B. Physicochemical Characterization of Ionic Liquid Binary Mixtures Containing 1-Butyl-3-Methylimidazolium as the Common Cation. *J. Chem. Eng. Data* **2019**, *64*, 4891–4903, doi:10.1021/acs.jced.9b00415.
34. Hiraga, Y.; Koyama, K.; Sato, Y.; Smith, R.L. High Pressure Densities for Mixed Ionic Liquids Having Different Functionalities: 1-Butyl-3-Methylimidazolium Chloride and 1-Butyl-3-Methylimidazolium Bis(Trifluoromethylsulfonyl)Imide. *J. Chem. Thermodyn.* **2017**, *108*, 7–17, doi:10.1016/j.jct.2016.12.037.
35. Machida, H.; Taguchi, R.; Sato, Y.; Smith, R.L. Measurement and Correlation of High Pressure Densities of Ionic Liquids, 1-Ethyl-3-Methylimidazolium L-Lactate ([Emim][Lactate]), 2-Hydroxyethyl-Trimethylammonium L-Lactate ([(C2H4OH)(CH3)3N][Lactate]), and 1-Butyl-3-Methylimidazolium Chloride ([Bmim][Cl]. *J. Chem. Eng. Data* **2011**, *56*, 923–928, doi:10.1021/je1008747.
36. Domańska, U.; Królikowska, M. Measurements of Activity Coefficients at Infinite Dilution in Solvent Mixtures with Thiocyanate-Based Ionic Liquids Using GLC Technique. *J. Phys. Chem. B* **2010**, *114*, 8460–8466, doi:10.1021/jp103496d.
37. Domańska, U.; Marciniak, A. Activity Coefficients at Infinite Dilution Measurements for Organic Solutes and Water in the Ionic Liquid 1-Butyl-3-Methylimidazolium Trifluoromethanesulfonate. *J.*

Phys. Chem. B **2008**, *112*, 11100–11105, doi:10.1021/jp804107y.

38. Domańska, U.; Wlazło, M.; Karpińska, M. Activity Coefficients at Infinite Dilution of Organic Solvents and Water in 1-Butyl-3-Methylimidazolium Dicyanamide. A Literature Review of Hexane/Hex-1-Ene Separation. *Fluid Phase Equilib.* **2016**, *417*, 50–61, doi:10.1016/j.fluid.2016.02.004.
39. Moïse, J.-C.; Mutelet, F.; Jaubert, J.-N.; Grubbs, L.M.; Acree, W.E.; Baker, G.A. Activity Coefficients at Infinite Dilution of Organic Compounds in Four New Imidazolium-Based Ionic Liquids. *J. Chem. Eng. Data* **2011**, *56*, 3106–3114, doi:10.1021/je200195q.
40. Domańska, U.; Królikowski, M. Determination of Activity Coefficients at Infinite Dilution of 35 Solutes in the Ionic Liquid, 1-Butyl-3-Methylimidazolium Tosylate, Using Gas-Liquid Chromatography. *J. Chem. Eng. Data* **2010**, *55*, 4817–4822, doi:10.1021/je100410k.
41. Lukoshko, E.; Mutelet, F.; Domanska, U. Experimental and Theoretically Study of Interaction between Organic Compounds and Tricyanomethanide Based Ionic Liquids. *J. Chem. Thermodyn.* **2015**, *85*, 49–56, doi:10.1016/j.jct.2014.12.027.
42. Ge, M.L.; Song, X.J.; Li, G.M.; Li, Y.H.; Liu, F.Z.; Ma, H.L. Activity Coefficients at Infinite Dilution of Alkanes, Alkenes, and Alkyl Benzenes in 1-Butyl-3-Methylimidazolium Dibutylphosphate Using Gas-Liquid Chromatography. *J. Chem. Eng. Data* **2012**, *57*, 2109–2113, doi:10.1021/je201310d.
43. Verevkin, S.P.; Sazonova, A.Y.; Frolkova, A.K.; Zaitsau, D.H.; Prikhodko, I. V.; Held, C. Separation Performance of BioRenewable Deep Eutectic Solvents. *Ind. Eng. Chem. Res.* **2015**, *54*, 3498–3504, doi:10.1021/acs.iecr.5b00357.
